# Branching topology of the human embryo transcriptome revealed by Entropy Sort Feature Weighting

**DOI:** 10.1242/dev.202832

**Published:** 2024-06-13

**Authors:** Arthur Radley, Stefan Boeing, Austin Smith

**Affiliations:** ^1^ Living Systems Institute, University of Exeter, Stocker Road, Exeter EX4 4QD, UK; ^2^ Bioinformatics and Biostatistics Science Technology Platform, The Francis Crick Institute, London NW1 1AT, UK

**Keywords:** Feature selection, Human development, Single cell transcriptomics, Continuous entropy sort feature weighting, Pluripotent stem cell, Epiblast

## Abstract

Analysis of single cell transcriptomics (scRNA-seq) data is typically performed after subsetting to highly variable genes (HVGs). Here, we show that Entropy Sorting provides an alternative mathematical framework for feature selection. On synthetic datasets, continuous Entropy Sort Feature Weighting (cESFW) outperforms HVG selection in distinguishing cell-state-specific genes. We apply cESFW to six merged scRNA-seq datasets spanning human early embryo development. Without smoothing or augmenting the raw counts matrices, cESFW generates a high-resolution embedding displaying coherent developmental progression from eight-cell to post-implantation stages and delineating 15 distinct cell states. The embedding highlights sequential lineage decisions during blastocyst development, while unsupervised clustering identifies branch point populations obscured in previous analyses. The first branching region, where morula cells become specified for inner cell mass or trophectoderm, includes cells previously asserted to lack a developmental trajectory. We quantify the relatedness of different pluripotent stem cell cultures to distinct embryo cell types and identify marker genes of naïve and primed pluripotency. Finally, by revealing genes with dynamic lineage-specific expression, we provide markers for staging progression from morula to blastocyst.

## Teaser

**Summary:** This study generates a high-resolution map of day 3-14 human embryo development by applying a new Entropy Sorting-based feature selection technique to single cell RNA-sequencing data.

## INTRODUCTION

Single cell RNA-sequencing (scRNA-seq) was first described in 2009 (Tang et al., 2009) and has since become a cornerstone of stem cell and developmental biology research. scRNA-seq in principle allows the expression levels of the entire transcriptome of individual cells to be measured in an unbiased manner. The mRNA profiles can then be used as a proxy to define the identity of each cell. A key application is to assess how transcriptomes change as cells transition from one cell state to another and thereby obtain insights into regulatory genes and networks.

Computational tools have been developed to extract useful information from the large and complex datasets that scRNA-seq generates. These bioinformatics pipelines can be broadly broken down into three main steps (Haque et al., 2017): (1) sequence alignment; (2) counts matrix processing; (3) data analysis. Here, we focus on the counts matrix processing stage of scRNA-seq analysis, which is a crucial step for increasing ability to extract biologically relevant insights from the data. Due to the importance of counts matrix processing, several open-source packages have been released. Notably, Seurat, Scanpy and Scran (Heumos et al., 2023) provide easy to use workflows for counts matrix processing, which can be further divided into three stages. Basic quality control removes low quality cells (low counts per cell, low genes per cell, high mitochondrial counts) and genes expressed in a very low number of cells, etc. Feature selection allows the identification of a subset of genes that are believed to be more informative of cell identity. Data denoising applies methods such as principal component analysis (PCA) and data regression/smoothing to mitigate the presence of artefacts such as technical noise and batch effects.

The primary aim of feature selection is to address the curse of dimensionality (Bellman, 1957) by finding a subset of the genes that are more discriminative of cell identity. In conventional workflows, this is typically achieved by selection of highly variable genes (HVGs). However, poor reproducibility between different methodologies has brought into question the robustness of HVG selection (Yip et al., 2019).

Following HVG selection, methods that augment or transform the data, such as feature extraction, data smoothing and batch integration, are employed to maximise the biologically relevant information that can be extracted (Chu et al., 2022). Although there are many examples of this approach successfully aiding the identification of interesting gene expression profiles, any computational technique that changes/augments the values in the counts matrix before downstream analysis has the potential to introduce artefacts. For example, smoothing techniques that try to impute and/or repair spurious gene expression values in a scRNA-seq counts matrix based on cell-cell similarities or gene-gene relationships may introduce computational false-negative or false-positive expression values that obfuscate real biological signals of interest (Andrews and Hemberg, 2018).

We sought to evaluate whether an alternative method of feature selection could increase the biological signal-to-noise ratio in scRNA-seq data in a manner that mitigates or removes the need for further counts matrix processing via feature extraction (e.g. PCA), data smoothing or batch integration. A key benefit of reducing data augmenting processes is that we may be more confident that patterns identified in further downstream analysis are not due to the introduction of computational artefacts.

We recently outlined a mathematical framework termed Entropy Sorting (ES) and incorporated it into two software packages, Functional Feature Amplification Via Entropy Sorting (FFAVES) and Entropy Sort Feature Weighting (ESFW) (Radley et al., 2023). Together, FFAVES and ESFW seek to identify genes that are highly structured within the data and are therefore more likely to be predictive of cell identity. Gene selection using these tools exposed the hitherto ambiguous inner cell mass (ICM) population in human pre-implantation embryo data (Radley et al., 2023; David et al., 2023). However, FFAVES and ESFW operate on binarised data, such that genes are considered active or inactive in individual cells. FFAVES is used to correct sub-optimal gene discretisation before the application of ESFW, which assigns feature importance weights. The ESFW weights are then used to select for a subset of all the genes in the data that are more informative of cell identity, in a similar manner to HVG selection.

Here, we update ES so that it can be applied to continuous data. We formulate this into a new software package, continuous Entropy Sort Feature Weighting (cESFW) and outline a workflow that uses cESFW to perform feature selection on scRNA-seq counts matrices. We validate cESFW on synthetic data and then apply the cESFW workflow to human embryo scRNA-seq datasets, achieving high resolution of cell types and developmental trajectories without augmentation or smoothing of the original data. Our analysis provides clear evidence for the two-step model of lineage segregation (Cockburn and Rossant, 2010) in early human embryo development by elucidating distinct branch points from morula to ICM or trophectoderm (TE), and from ICM to epiblast (Epi) or hypoblast (Hyp).

These findings demonstrate that cESFW can reveal gene expression states and transitions in scRNA-seq data that were previously unobservable through conventional feature selection techniques.

## RESULTS

### Proposed cESFW feature selection workflow for scRNA-seq data

**Fig. 1.**
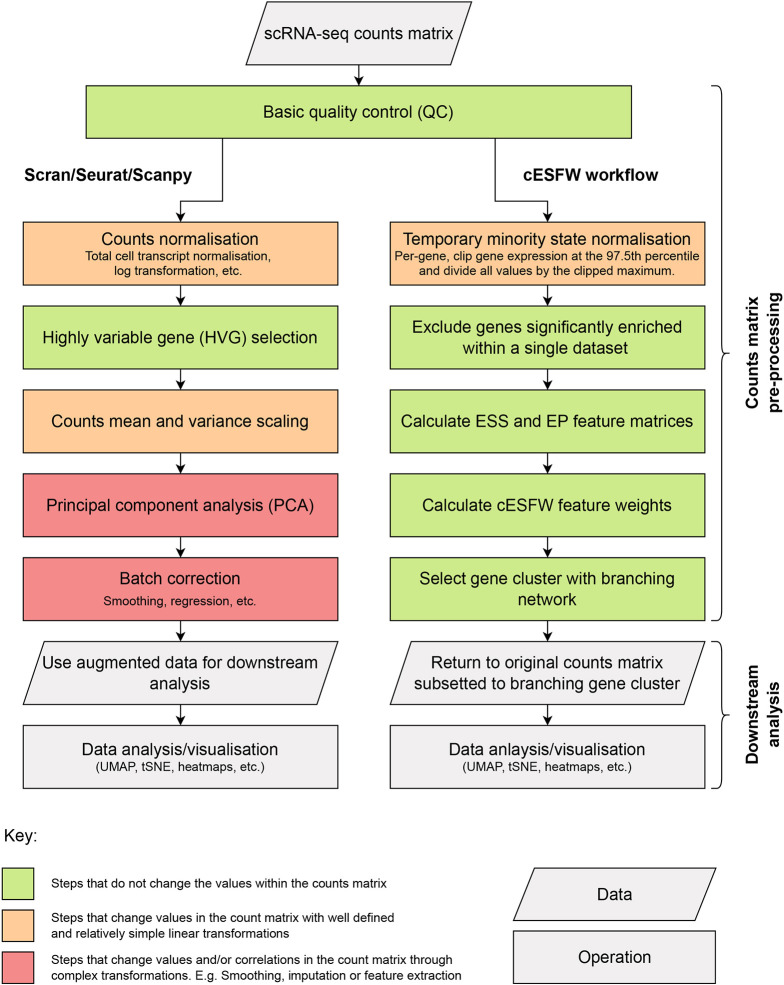
**scRNA-seq counts matrix processing workflows.** Left hand column shows a high-level outline of the workflows implemented by widely used scRNA-seq bioinformatics pipelines such as Scran, Seurat and Scanpy. Right hand column outlines the steps of our cESFW workflow. Colours indicate the degree to which the steps alter expression counts values in the original scRNA-seq counts matrix before the data are passed onto downstream analysis steps.

Data analysis packages such as Seurat, Scanpy and Scran involve multiple steps, some of which alter the values in the original counts matrix. In Fig. 1, HVG selection is highlighted in green because, although it reduces the size of the counts matrix by subsetting down to a smaller set of genes, none of the expression values within the matrix is changed. Conversely, PCA and batch correction methods are highlighted in red because they augment the starting scRNA-seq counts matrix before passing the data onto the next step of analysis, and hence have a potential to introduce computational artefacts.

As an alternative to conventional workflows, we propose cESFW (Fig. 1). This workflow facilitates unsupervised gene importance weighting based on entropy sorting rather than HVG selection. Below, we demonstrate that the cESFW gene selection methodology leads to improved downstream analysis, without the need to change any of the expression values in the original counts matrix. This reduces the potential for computational artefacts to be introduced into the analysis of an scRNA-seq dataset. A key advance from our previous approach (Radley et al., 2023) is that cESFW can be applied to data with continuous values. For a description of how ESFW was adapted into cESFW see supplementary Materials and Methods, and for a detailed breakdown of each step of our cESFW workflow see Materials and Methods.

### cESFW robustly discriminates between significant and non-significant gene correlation signals in synthetic data

The foundation of the cESFW workflow is the use of cESFW to perform feature selection in place of HVG feature selection. Both cESFW and HVG feature selection aim to take a high dimensional scRNA-seq dataset and subset it down to a set of genes that are believed to be more informative of cellular identity than the entire gene set. We used synthetic data to perform a side-by-side analysis of cESFW and the HVG implementations of Scran and Seurat. The Scanpy protocol was excluded because, rather than providing a ranked list of genes, Scanpy provides a true/false index for selected HVGs, making it difficult to quantify performance.

**Fig. 2.**
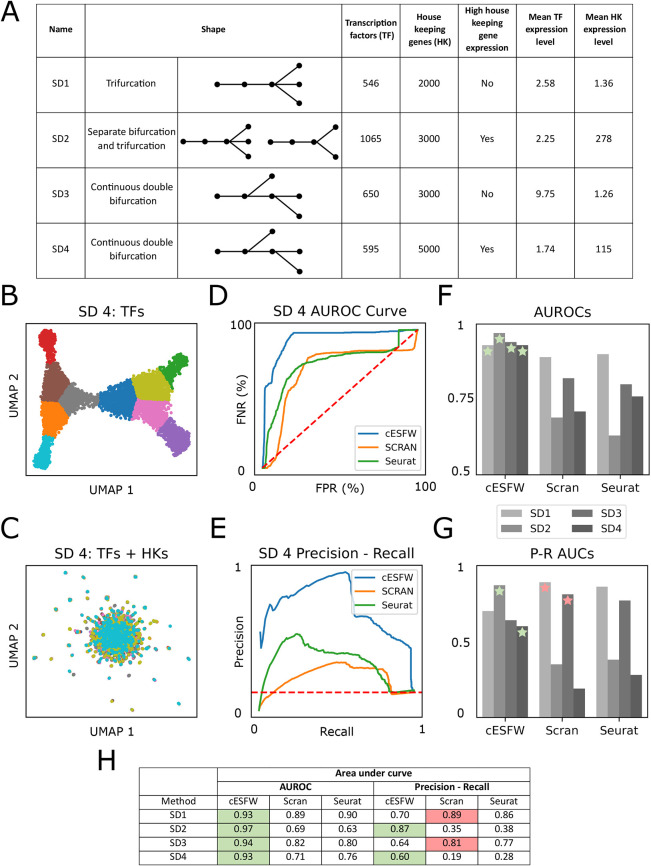
**Comparison of cESFW and HVG feature selection techniques.** (A) Summary of the four synthetic datasets (SD) of varying size and shape created using the Dyngen software. (B) UMAP of SD4 created using only the transcription factor (TF) genes. (C) UMAP of SD4 created using all genes [TFs and housekeeping (HKs)]. (D) AUROC curve generated using the ranked gene lists from application of cESFW, Scran and Seurat gene selection to SD4. (E) Precision-Recall curve using the same inputs as D. (F) AUROC scores after applying cESFW, Scran and Seurat gene selection to all four SDs. (G) Precision-Recall AUCs using the same inputs as F. Green stars indicate that cESFW had the highest score for an SD and red stars indicate when a different method had a higher score than cESFW. (H) Summary of all area under curve (AUC) results. Green boxes indicate that cESFW had the highest score for an SD and red boxes indicate when a different method had a higher score. For UMAPs and AUC curves relating to SD1-SD3, see Fig. S1.

We used Dyngen (Cannoodt et al., 2021) to create four synthetic datasets (SD) (Fig. 2A) with known ground truths as to which genes relate to cell-state-specific transcription factor (TF) networks and which are part of ubiquitous housekeeping (HK) gene networks. Dyngen generates synthetic scRNA-seq data by simulating empirically derived gene regulatory networks (GRNs), while allowing control over the size and shape of the generated data. The column labelled ‘High housekeeping gene expression’ (Fig. 2A) refers to a property of Dyngen such that at different random seeds, the average expression of the simulated genes can vary significantly. We took advantage of this to intentionally find random seeds that generated data where either the HK genes had expression levels comparable with the TFs (High housekeeping gene expression=No) or the HK genes had relatively high expression levels compared with the TFs (High housekeeping gene expression=Yes). For example, the average non-zero expression of SD3 TFs and HKs are 9.75 and 1.26, respectively, whereas for SD4 they are 1.74 and 115, respectively (Fig. 2A). We introduced this layer of analysis because previous evaluations of HVG selection methods have highlighted that the gene selection processes can be biased towards genes that have higher mean expression (Yip et al., 2019). The effect of highly expressed HK genes is shown in Fig. 2B,C. In Fig. 2B, the UMAP generated for SD4 using just the TFs shows the expected branching trajectories, whereas in Fig. 2C, including the HK genes for UMAP generation obscures the branching gene expression dynamics.

Dyngen allows us to differentiate between genes informative of cell type (TFs) and ubiquitously expressed genes (HKs). We used this to quantify the performance of the ranked gene lists generated by cESFW, Scran and Seurat using receiver operating characteristic (ROC) and Precision-Recall (PR) curves (Grau et al., 2015). For this analysis, the area under (AU) the ROC and PR curves give scores between 0 and 1, where 1 means that the feature selection method is perfect at discriminating between cell type informative TF genes and non-informative HK genes. We show both the ROC and PR curves (Fig. 2D,E) because ROC is more readily comparable across datasets, whereas PR provides a comparable metric of method performance that is more sensitive to class imbalance (many more HK genes compared with TF genes). The AUROC and PR curves for SD4 are shown in Fig. 2D,E, respectively. In both cases, the area under the blue cESFW curve is largest, indicating that cESFW outperforms Scran or Seurat at ranking TFs as more informative of cell identity than HKs. The results of all the ROC and PR curves are summarised in Fig. 2F-H. The AUROC scores of cESFW are higher than Scran and Seurat on all four SDs (Fig. 2F). High AUROC scores can be thought of as enriching highly ranked genes with genes that are indicative of cell type.

Although cESFW does not have the highest PR-AUCs score for SD1 and SD3 (Fig. 2G; Fig. S1C,D), we note that the drop in performance is less than the gain for SD2 and SD4. As SD2 and SD4 are the datasets in which HK gene expression is relatively high compared with TF expression, this reinforces previous findings that HVG selection can be biased towards highly expressed genes (Yip et al., 2019). Therefore, the results in Fig. 2G suggest that cESFW is less sensitive to the presence of highly expressed genes than HVG selection methods.

These results on four independent SDs show that cESFW can provide more robust feature selection than HVG selection.

### cESFW reveals high resolution gene expression dynamics in the early human embryo

We incorporated cESFW into the proposed workflow (Fig. 1, right hand column) and exemplified application to biological scRNA-seq using a reference dataset of peripheral blood mononuclear cells (PBMCs) (Stuart et al., 2019). We saw that cESFW performed comparably to Seurat and Scran in identifying cell types and more stably maintains cell type clusters with increasing number of selected top ranked genes (Fig. S2).

We next applied the cESFW workflow to six merged independent scRNA-seq datasets of early human embryo development (Yan et al., 2013; Fogarty et al., 2017; Petropoulos et al., 2016; Yanagida et al., 2021; Meistermann et al., 2021; Xiang et al., 2020). These datasets have previously been characterised (Petropoulos et al., 2016; Stirparo et al., 2018; Meistermann et al., 2021; Singh et al., 2023; Wei et al., 2023), and there is a foundation of experimental knowledge regarding the expected distinct cell types and stages.

**Fig. 3.**
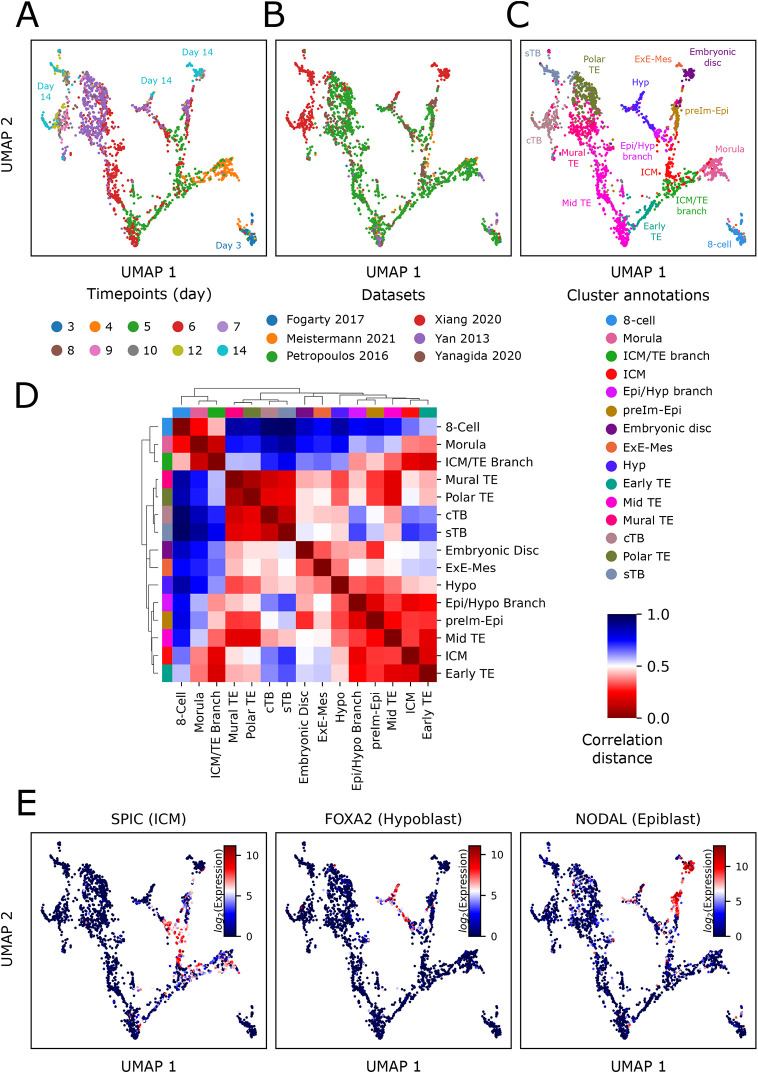
**High-resolution UMAP embedding of early human embryo development.** (A-C) UMAP embedding generated using the 3012 genes identified by our cESFW workflow coloured by time point labels from data source papers (A), coloured by dataset sources (B) and coloured by our cell state cluster annotations (C). cTB, cytotrophoblast; Epi, epiblast; ExE-Mes, extra-embryonic mesenchyme; Hyp, hypoblast; ICM, inner cell mass; preIm-Epi, pre-implantation epiblast; sTB, syncytiotrophoblast; TE, trophectoderm. See Figs S3 and S4 for further details regarding the cluster annotations. (D) Heatmap showing similarity of annotated cell states by calculating the correlation distance matrix of pseudobulk samples using the 3012 cESFW selected genes. Low correlation distances (red) indicate similar samples. (E) Known cell type marker expression profiles validate our UMAP annotations. See Fig. S4 for more examples.

The cESFW workflow identified a set of 3012 genes. For details and code see Materials and Methods. Gene set enrichment analysis found Gene Ontology (GO) terms relating to regulation and development. Using this gene set we obtained a smooth, high resolution uniform manifold approximation and projection (UMAP) embedding (Fig. 3A; Fig. S3A). Importantly, the UMAP embedding was generated by simply subsetting down to the 3012 cESFW workflow genes, without any data augmentation or smoothing. The observed structure should therefore reflect biologically significant gene expression patterns, free of computational artefacts that may be introduced through conventional scRNA-seq counts matrix processing workflows (Fig. 1, left hand column). The UMAP clearly displays continuous progression along the developmental time course. Cells from the five pre-implantation embryo datasets are intermingled across the UMAP space, indicating minimal contribution of batch effects (Fig. 3B; Fig. S3B). Cells from the post-implantation extended culture samples (Xiang et al., 2020) form groups adjacent to distinct regions of pre-implantation cells, indicating that related gene expression patterns have been identified across the pre- and post-implantation datasets.

We performed unsupervised agglomerative clustering on the scRNA-seq counts matrix subset down to the 3012 cESFW genes. Overlaying these clusters onto the UMAP embedding (Fig. S3A) provided an unsupervised foundation to label samples with 15 distinct cell type/stage annotations (Fig. 3C; Fig. S3B,C). Comparison with annotations from previous studies (Petropoulos et al., 2016; Stirparo et al., 2018) corroborated our annotations and UMAP embedding (Fig. S3D,E). Notably, the unsupervised clusters were readily annotated, whereas previous annotations were generated through supervised analysis focused on a handful of candidate marker genes. A heatmap of pseudobulk samples generated by taking the mean expression levels of genes for samples in each of our annotated cell states reveals how closely cell populations are related to one another (Fig. 3D). Genes implicated in the literature to correspond with 13 of our cell state annotations are represented in the 3012 cESFW gene set (Cockburn and Rossant, 2010; Taubenschmid-Stowers et al., 2022; Stirparo et al., 2018; Singh et al., 2023; Corujo-Simon et al., 2023; Liu et al., 2022; Zadora et al., 2017; Yue et al., 2020; Yabe et al., 2016; Yang et al., 2021). Such genes show specific gene expression in the UMAP embedding (Fig. 3E; Fig. S4). Mural TE lacks validated specific markers but is known to express HAND1 (Liu et al., 2022). Although HAND1 is not captured in the cESFW gene set, it shows relatively specific day 6/7 mural TE expression on our UMAP embedding (Fig. S4).

Visualisation of chronological embryo progression with gene expression patterns consistent with the literature demonstrates that the cESFW workflow identifies a set of genes that is highly descriptive of the transcriptional landscape of day 3-14 developing human early embryos. We have created an interactive online portal for visualisation of gene expression profiles on our UMAP embedding and generation of plots at https://bioinformatics.crick.ac.uk/shiny/users/boeings/radley2024umap_app/.

#### Comparison of cESFW with HVG selection techniques on human embryo data

To compare the performance of cESFW with HVG selection using the same input data, we identified the 3012 top ranked genes for Scran and Seurat workflows. Previous studies comparing HVG selection methods have shown that there is often poor concordance between gene sets selected by different methods (Yip et al., 2019). Intersecting the gene sets from the cESFW, Scran and Seurat workflows, we found only 290/3012 (9.63%) are common to all three (Fig. S5A). We then compared UMAP embeddings. For a fair comparison, we omitted prior PCA selection for the Scran and Seurat UMAPs, as PCA reduction is not part of the cESFW workflow. Scran and Seurat UMAPs broadly separated samples by embryo time points but did not provide a smooth embedding with a coherent progression of cell states and developmental trajectories (Fig. S5C,D).

We used silhouette scores to quantify how well each of the 3012 gene sets formed distinct clusters of samples, using our UMAP cell state annotations (Fig. 3C) as cluster labels. Silhouette scores measure how similar samples are to their designated clusters, with positive scores indicating samples are most similar to the cluster they are a member of, and negative scores designating samples that would be better assigned to a different cluster. The average of the silhouette scores for all samples within a cluster then quantifies the quality of the cluster as whole. The cESFW genes produced higher silhouette scores than the Seurat or Scran gene sets for 14 out of the 15 clusters, and similar scores for ICM (Fig. S5B). Notably the Epi/Hyp branch, Hyp and Mid TE score positive with cESFW but have negative silhouette scores using HVG, signifying that the HVG gene sets are unable to identify these cell types as distinct from other cells in the data.

These results show qualitatively and quantitatively that cESFW gene selection outperforms HVG selection workflows on these human embryo scRNA-seq data and help explain why the UMAP generated using the 3012 cESFW gene set is better able to readily separate known cell types from one another.

### Lineage branching during blastocyst development

The textbook model of blastocyst formation derived from studies in the mouse embryo entails sequential lineage bifurcations that generate first TE and ICM, and then Hyp and Epi (Cockburn and Rossant, 2010). However, the existence of two branch points in human embryogenesis has been questioned in analyses of scRNA-seq data (Petropoulos et al., 2016; Goedel and Lanner, 2021). More recent studies have proposed two bifurcations, but have not demonstrated separate branch point populations (Yanagida et al., 2021; Radley et al., 2023; Wei et al., 2023).

**Fig. 4.**
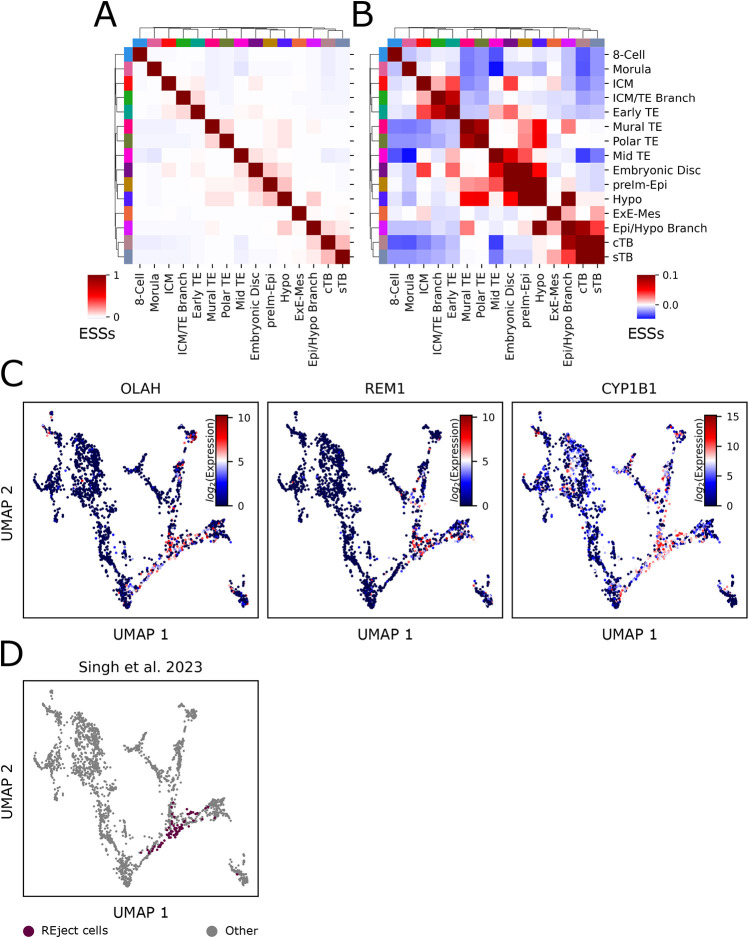
**REject cells are within the ICM/TE branch point cluster.** (A,B) Quantification of cell state co-occurrence in individual embryos. See Materials and Methods for details. Heatmap in A shows cell state co-occurrence in individual embryos, quantified via ESSs. Hierarchical clustering shows that temporally close cell states co-occur with each other most frequently. Colour bar shows ESSs. B shows the same as A, but the colour bar has been truncated so that the maximum value is 0.1, to better appreciate cell co-existence in individual embryos. (C) Proposed ICM/TE branch marker genes. cTB, cytotrophoblast; ExE-Mech, extra-embryonic mesenchyme; Hypo, hypoblast; ICM, inner cell mass; preIm-Epi, pre-implantation epiblast; sTB, syncytiotrophoblast; TE, trophectoderm. (D) REject cells identified by Singh et al. (2023) coloured on human embryo UMAP from Fig. 3.

The cESFW embedding unambiguously reveals two binary branch points in pre-implantation embryogenesis: from morula to ICM or early TE, and from ICM to Epi or Hyp. Unsupervised clustering identifies populations of cells at these intersections which we labelled ICM/TE and Epi/Hyp branch cells, respectively (Fig. 3C). Silhouette scores quantify both as distinct groups of cells according to transcriptional profile (Fig. S5B). Examining the co-occurrence of cell states in individual embryos further substantiates the two step model (Fig. 4A,B; Fig. S6A,B). ICM/TE branch and early TE cells most commonly co-occur with one another, whereas ICM cells rarely co-exist in embryos with Epi or Hyp. In Fig. 4C, we used our UMAP and ranked gene list (Table S1) to highlight three genes that show relatively specific upregulation within the ICM/TE branch population.

To examine the inferred lineage trajectories, we performed RNA velocity analysis (Bergen et al., 2020) (Fig. S6D) and trajectory inference using the STREAM software package (Chen et al., 2019) (Fig. S6E). The RNA velocity and trajectory inference results support the existence of branch points between TE and ICM fates, and ICM to Epi/Hyp fates. The RNA velocity vectors are aligned well with temporal ordering and lineage divergences across the embedding.

In a recent publication, Singh et al. (2023) re-analysed the Petropoulos et al. (2016) human embryo scRNA-seq dataset and highlighted a previously undescribed group of day 4/5 cells. These authors were unable to place this cluster in a differentiation trajectory and noted upregulation of several proposed apoptosis markers. They therefore speculated that that this group of cells had accumulated DNA damage and was fated to apoptose, denoting them ‘REject’ cells. However, when we inspected the REject cells in the cEFSW embedding we found that they do not constitute a separate cluster but are almost entirely within the ICM/TE branch point (Fig. 4D). We therefore investigated the markers suggested to indicate that REject cells are pre-apoptotic. We found that most of these genes are expressed as highly elsewhere in the developing human embryo, suggesting either that they are not strong discriminators of pre-apoptotic versus non-apoptotic cells, or that apoptosis is distributed across cell types (Fig. S7). For example, although suggested apoptosis markers *Bik*, *Atg2a* and *Atf3* are upregulated in the ICM/TE branch when compared against the early TE, ICM, pre-implantation Epi (preIm-Epi) and Hyp populations, these three genes are expressed more highly in the eight-cell and/or morula populations (Fig. S7B). In a similar vein, *Bak1*, *Ctsb* and *Casp6* were suggested to be apoptotic markers specific to the REject population, but we found these genes to be broadly expressed across several cell types of day 3-14 human embryos (Fig. S7C). Thus, we did not find strong indications that the proposed REject cells are specifically fated for apoptosis.

Overall, these findings substantiate the occurrence of an initial lineage bifurcation between TE and ICM on day 4/day 5 that precedes segregation of ICM to Hyp and Epi between days 5 and 6.

### Comparison of human pluripotent stem cell cultures to reference human embryo scRNA-seq embedding

**Fig. 5.**
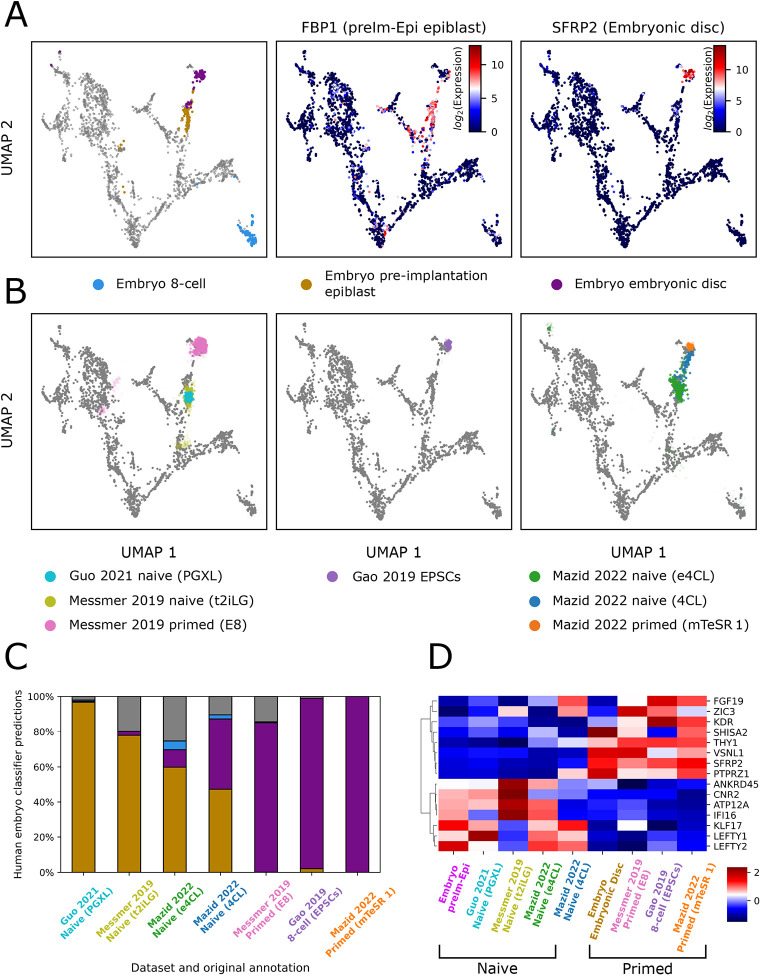
**Characterising the human embryo counterparts of *in vitro* stem cell cultures.** (A) UMAP embedding highlighting the embryo eight-cell, pre-implantation epiblast (preIm-Epi) and embryonic disc populations. FBP1 and SFRP2 shown as previously identified preIm-Epi and Embryonic disc markers, respectively (Messmer et al., 2019). (B) Projection of cultured naïve, primed and expanded potential stem cells (EPSCs) scRNA-seq datasets onto our UMAP embedding. To aid interpretation, sample transparencies are scaled by the density of samples on the embedding from the same condition, such that samples in the densest region are completely opaque. Abbreviations in brackets denote relevant culture media. (C) Quantification of cultured naïve, primed and EPSC scRNA-seq sample identities. Colour coding as in panel A, with grey denoting unassigned. (D) Heatmap of *z*-score normalised gene expression of eight naïve specific and eight primed specific gene markers. All values are pseudobulk expression values, generated by grouping single cells by their dataset and predicted cell state labels from panel C, and taking the average expression of each group.

Human pluripotent stem cells (PSCs) are generally considered to be analogues of the pluripotent epiblast lineage in the embryo. However, PSCs exist in different states that are propagated in distinct signalling environments (Smith, 2017; Nichols and Smith, 2009). Correlation of PSCs with developmental stages of Epi in the human embryo has been problematic due to the scarcity of relevant data and the limited resolution provided by previous analysis methods. We took the opportunity provided by our high-resolution embedding to re-evaluate the transcriptome relatedness of various cultures of human PSCs to cell types in the day 3-14 embryo, highlighting preIm-Epi and post-implantation Epi (postIm-Epi) (Fig. 5A; Fig. S4).

We examined published scRNA-seq datasets (Guo et al., 2021; Messmer et al., 2019) from conventional primed PSCs and cells in two naïve culture conditions, PXGL (Guo et al., 2017) and its predecessor t2iLGö (Takashima et al., 2014). We also assessed data for expanded potential stem cells (EPSCs), originally suggested to be related to the eight-cell stage (Gao et al., 2019). We used the umap.transform function of the Python UMAP package (McInnes et al., 2018) to map the query cells from the PSC cultures onto our reference human embryo UMAP. This method places individual query samples closest to their most transcriptionally similar counterparts in the UMAP in an unsupervised manner based on all 3012 cESFW genes. Fig. 5B shows the projections of individual cells from the query scRNA-seq datasets onto the embedding. Conventional primed PSCs are placed in the vicinity of the day 10-14 embryonic disc Epi. EPSCs are similarly positioned close to postIm-Epi, with *<*2% of cells adjacent to earlier stages. These findings are consistent with previous comparative analyses with non-human primate embryos (Guo et al., 2021; Nakamura et al., 2017). In contrast, naïve PSCs overlie preIm-Epi (day 6-7). Notably cells in PXGL are more homogeneous than cells in t2iLGö. Quantification using a k-nearest neighbour classifier trained on the embryo cell state annotations (Fig. 5C) assigns 97% of PXGL cells to preIm-Epi, compared with 77% for t2iLGö.

We also evaluated published data for 4CL and e4CL cells that are proposed to be related to day 5 ICM and eight-cell stages, respectively (Mazid et al., 2022). These samples are more heterogeneous than other PSC cultures. They comprise preIm-Epi-like proportions of 48% for 4CL and 60% for e4CL. A substantial fraction (39%) of 4CL cells are postIm-Epi-like, whereas many e4CL cells are positioned between preIm-Epi and postIm-Epi, indicating either an indeterminate mixed identity or transitioning intermediates. e4CL cultures contain only 5% of cells which are related to day 5 ICM and 5% of cells related to the eight-cell embryo.

To validate UMAP label transfer, we calculated the correlation distances between samples grouped by our embryo cell type annotations and each of the PSC scRNA-seq samples (Fig. S8). The results closely match those in Fig. 5C for all samples, demonstrating that label transfer in the low dimensional UMAP space is consistent with cell type prediction in the high dimensional 3012 gene space.

#### Identifying naïve and primed human PSC markers

Previous studies have identified gene markers for naïve and primed human PSCs by examining differential expression between these two PSC types specifically. We sought to characterise markers in the context of the entire day 3-14 human embryo. We started by taking the top three naïve versus primed PSC markers identified by Messmer et al. (2019) and plotted them on our UMAP embedding (Fig. S9). We saw that, although these six markers were clearly up- or downregulated between the preIm-Epi and embryonic disc samples, for five out of the six genes, expression is not restricted to the Epi lineage. In contrast, the top three ranked genes for our UMAP-annotated preIm-Epi and embryonic disc cell types (Table S1; Fig. 3C) show more restricted expression to the preIm-Epi or embryonic disc regions (Fig. S9).

We then proceeded to use our rank gene lists to identify genes that showed specific upregulation in the preIm-Epi or embryonic disc, while also showing differential expression in naïve versus conventional human PSCs. In Fig. 5D we present a heatmap of eight naïve and eight primed marker genes that have consistent cell-type-specific differential expression across the cell line datasets, and in our reference human embryo embedding. We also note that, in this heatmap, the 4CL cells show upregulation of both naïve and primed markers, consistent with evidence from UMAP label transfer and correlation analysis that this culture condition displays mixed identity (Fig. 5B,C; Fig. S8).

This analysis of naïve, primed and EPSC stem cells from different research groups demonstrates how access to an unbiased view of the early human embryo can facilitate classification of *in vitro* cell cultures. Furthermore, we show that taking a more holistic approach to cell state marker identification can provide more specific cell type marker genes.

### Emergence of Epi, Hyp and TE signatures during blastocyst formation

Between days 3 and 7 post fertilisation, the morula of the human embryo develops into the blastocyst, comprising three distinct lineages: Epi, Hyp and TE. We sought to use the cESFW embedding to identify genes that may be useful markers for staging cell identities during blastocyst formation.

The Entropy Sort Score (ESS), is a correlation metric derived from ES (Radley et al., 2023). A potential benefit of the ESS for gene ranking compared with other typical methods such as the *t*-test or Wilcoxon test is that the ESS was specifically derived to reduce whenever gene expression is observed outside of a specific population of interest. Therefore, when there are multiple cell types within a dataset, the ESS can be better suited for identifying regions of specific gene expression, as opposed to more general up- or downregulation. Using the ESS, we ranked genes by how well their expression profiles overlap with the Epi, Hyp or TE morula-to-blastocyst trajectories, while also progressively restricting the analysis to the terminal Epi, Hyp or TE populations. For examples and details regarding this progressive restriction, see Fig. S10 and Materials and Methods.

**Fig. 6.**
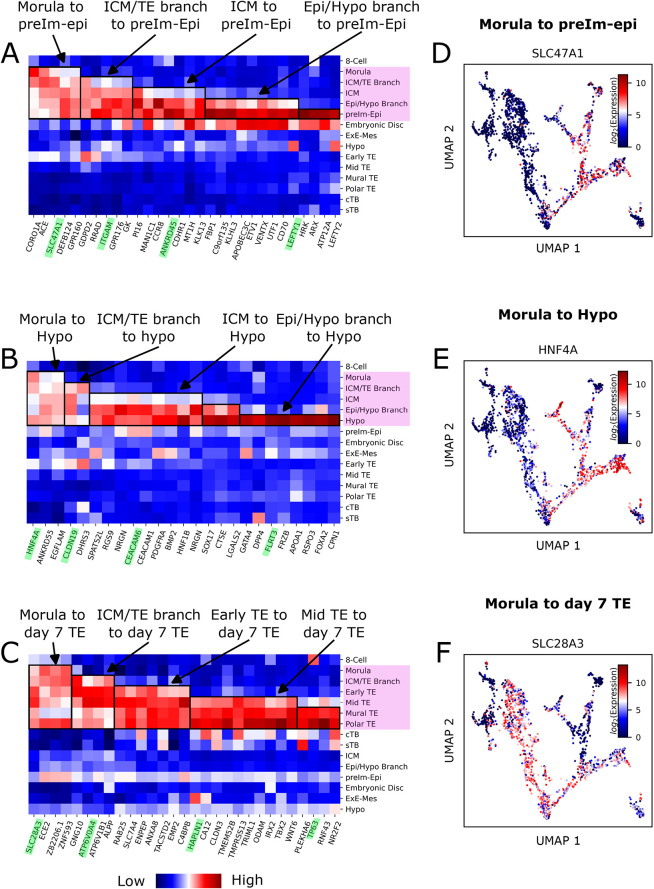
**Emerging epiblast, hypoblast and trophectoderm signatures during blastocyst development.** (A-C) Heatmaps of pseudobulk *z*-score-normalised human embryo data, displaying genes that show blocks of sequential upregulation between morula and Epi (A), Hyp (B) or TE (C) lineages. Pink boxes highlight most relevant cell types for each lineage. Green highlighted genes are presented in UMAPs within this figure and in Fig. S10. (D-F) UMAP gene expression profiles for example genes showing expression from morula to Epi (D), Hyp (E) or TE (F) lineages. Epi, epiblast; Hypo, hypoblast; ICM, inner cell mass; preIm-Epi, pre-implantation epiblast; TE, trophectoderm.

In Fig. 6A-C we present heatmaps highlighting sets of genes that show sequential upregulation as the morula differentiates into Epi (A), Hyp (B) or TE (C). Day 7 TE comprises both mural and polar TE. Fig. 6D-F and Fig. S10 show example genes with specific upregulation along each trajectory. For example, during the emergence of TE cells during blastocyst formation, *Slc28a3* (Fig. 6F) is first upregulated in the morula and maintains expression until day 7 TE, while being downregulated in the ICM, Epi and Hyp lineages. Conversely, the TE marker *Hapln1* (Fig. S10B) is upregulated later than *Slc28a3* at around day 6 and is maintained in day 7 of TE.

These findings illustrate how ES and our UMAP embedding can be used to identify new gene expression patterns that may aid future experiments and analyses, also exemplified in Dattani et al. (2024).

## DISCUSSION

In this work, we introduce cESFW, a feature selection method designed for distinguishing between cell state informative and uninformative genes in high dimensional scRNA-seq data. A key ethos of the cESFW workflow is that the original values in the scRNA-seq counts matrix are not changed in any way. We show that using cESFW to subset down to a set of informative genes is not only sufficient to identify dynamic gene expression states, but can outperform more complex methods that augment the values of the counts matrix. These findings challenge the view that scRNA-seq data are inherently too noisy to identify high resolution gene expression dynamics without repairing, smoothing or transforming the data first.

On synthetic scRNA-seq data we found that cESFW performs comparably with or better than conventional HVG selection. HVG selection has been shown to be sensitive to the presence of highly expressed genes, which can lead to a significant drop in feature selection performance. Previous work has shown that Seurat and Scran HVG selection are more robust to differences in mean expression than other HVG selection methodologies (Yip et al., 2019). However, on our synthetic data we found that both Scran and Seurat HVG selection performance dropped significantly when data contained a large number of highly-expressed HK genes. Conversely, cESFW feature selection is relatively agnostic to the presence of genes with high mean expression, with a minimal drop in performance. Less influence of expression level when ranking gene importance may be a significant contributor to the superior performance of cESFW on biological data. In addition, the multivariate approach of cESFW may be more robust than univariate HVG feature selection. In the univariate approach, genes are considered in isolation, leading to their importance ranking being more sensitive to fluctuations in properties such as mean expression. cESFW, in contrast, considers pairwise the expression profile of an inspected gene with every other gene in the data, and generates a gene importance weight by quantifying the degree to which the inspected gene forms networks of significantly co-expressing genes.

Applying our cESFW workflow to early human embryo scRNA-seq data generates a high resolution UMAP embedding of different cell states, trajectories and branch points in the developing embryo. Our cell state annotations formed quantifiably more distinct clusters when using top rank genes selected by cESFW compared with the top ranked genes from conventional HVG selection. Furthermore, we obtain a coherent merged dataset without having to apply batch correction methods as in previous analyses (Meistermann et al., 2021; Wei et al., 2023). We propose that cESFW is effective for combining datasets from different sources because improved feature selection tips the biological signal-to-noise ratio in favour of the biologically relevant expression profiles in the data. Cell similarity metrics may thus be less affected by confounding gene expression signals.

Our high-resolution map of the early human embryo transcriptome allowed us to substantiate the existence of two distinct branching populations, at the intersections of morula with ICM and TE, and of ICM with Epi and Hyp. Their identification and the general topology of the cESFW embedding indicate that human embryogenesis proceeds via the sequence of lineage bifurcations established for the mouse embryo (Cockburn and Rossant, 2010).

The branching populations reside at crucial junctures in blastocyst formation, the partitioning of extra-embryonic and embryonic lineages. These branch point clusters do not define unitary states. On the contrary, cells in these clusters are heterogeneous and may become specified to alternative fates. For example, PDGFRA, a Hyp marker (Corujo-Simon et al., 2023), and NANOG, an Epi marker (Allègre et al., 2022), are heterogeneously distributed in the Epi/Hyp branching population. Furthermore, branch cluster boundaries extend beyond the bifurcation region, potentially indicating that cells remain plastic and may be redirected. Notably, it has been seen in mouse embryos that cells expressing ICM genes remain capable of generating TE up to the late 32-cell stage (Posfai et al., 2017). Further analytical and experimental studies may focus on heterogeneity and gene expression dynamics in these branch point populations to unravel the process of lineage choice and commitment.

Our analyses call for a reappraisal of the proposal that the human embryo contains a distinct population of cells that are excluded from development and fated for apoptosis (Singh et al., 2023). We found that so-called REject cells are located on the developmental trajectory, residing in the ICM/TE branch population. Those cells do express some markers of a pre-apoptotic state, but so do other cells throughout the data. Quantitative immunostaining analyses are needed to reveal whether there is a consistently higher frequency of apoptosis in this population in the embryo.

We used the cESFW gene set and embedding as a reference point to evaluate the identity of various human PSC cultures. Quantifying proportional relatedness of cell lines to embryo populations confirmed that conventional human PSCs and EPSCs are most related to post-implantation embryonic disc, whereas naïve PSCs are similar to preIm-Epi. This analysis also demonstrated that PXGL naïve PSCs have a very high (97%) representation of preIm-Epi-like cells.

Finally, we used ESSs and our UMAP embedding to suggest sets of genes that may aid cell type staging during days 3-7 of lineage progression during human blastocyst formation. Immunostaining to assess protein expression dynamics for these genes will be of interest.

In summary, this study demonstrates that cESFW, an algorithm derived from the mathematical framework of ES, has the ability to reveal gene expression dynamics in scRNA-seq data that were previously unobserved. The expression profiles of each of 16,719 genes available in the scRNA-seq counts matrix can be visualised and plotted at the online portal https://bioinformatics.crick.ac.uk/shiny/users/boeings/radley2024umap_app/. Our characterisation of early human embryo scRNA-seq data has provided ranked gene lists for multiple cell states and a high resolution UMAP embedding. We provide a proposed workflow for incorporating cESFW into general scRNA-seq analysis. With wider application, we expect to gain greater insights into which scenarios cESFW can outperform or complement conventional HVG selection. We propose that cESFW may be particularly suited to analysis of transcriptome dynamics and trajectories in time course series or during developmental progression. We also envisage that the cESFW technique and workflow will be readily adapted for application to datasets of varying complexity and type, such as multi-omics or spatial transcriptomics studies.

### Limitations of the study

As more embryo scRNA-seq datasets become publicly available, re-analysis should yield greater resolution. A higher density of samples from day 4-6 embryos will be valuable for better delineation of ICM progression and lineage branch points. Further data from extended cultures will enable greater resolution of embryonic disc progression and emergence of extra-embryonic mesenchyme and amnion.

A ground truth is lacking for human embryo scRNA-seq. To quantify the performance of Seurat and Scran compared with cESFW feature selection, we took the top 3012 genes from the ranked gene lists and calculated the cluster Silhouette scores based on our cell state annotations. We chose to use our cluster annotations because we believe we have provided substantial evidence of the biological relevance. However, this approach may introduce some bias towards cESFW.

The genes we have highlighted for staging of cells during blastocyst formation were chosen through semi-supervised analysis. The initial gene sets were curated from ranked gene lists output based on the ESS. However, the final sets of selected genes were subject to manual inspection of the top 30 ESS ranked genes, and genes that appeared to be poorly expressed in the cell populations of interest were manually excluded. Therefore, the presented gene sets may be incomplete.

Finally, the benefits of feature selection through cESFW do not preclude the use of other data analysis techniques such as batch integration and data repair. We suggest that more accurate feature selection has the potential to improve results of all downstream steps. We anticipate adaptations of our proposed cESFW workflow that incorporate a range of data analysis methodologies.

## MATERIALS AND METHODS

### From discrete data Entropy Sorting to continuous data Entropy Sorting

A key distinction between the ESFW algorithm outlined in our previous work (Radley et al., 2023) and the cESFW algorithm presented in this work is that cESFW updates the theory behind ES so that it can be readily applied to continuous data. For a detailed explanation of how ES has been adapted for continuous data, see supplementary Materials and Methods.

### A fifth entropy sorting error scenario

In addition to expanding the ES framework so that it may be applied to continuous data, we also introduce a fifth error scenario where ES divergence can be observed through the ES parabola in a manner that indicates that samples may be displaying false negative (FN) expression levels. We present this new error scenario in Fig. S11 (black box), alongside the four maximum entropy error scenarios introduced in our previous manuscript (Radley et al., 2023). The error scenarios are important because they provide implicit logic that determines how we calculate divergence and error potential for any pair of features.

As an example, in our newly proposed fifth error scenario, there are two features being compared against one another. The top feature (blue and red) is denoted the reference feature (RF) and the bottom feature (green and purple) is denoted the query feature (QF) because, according to the maximum entropy principle, the QF should always be the feature with the larger cardinality for its observed minority state (Radley et al., 2023). For this pair of features, we see a strong but imperfect conditional relationship between the co-occurrence of ground truth (absent of X symbols) RF and QF minority states. However, the presence of RF majority states overlapping with QF minority states introduces uncertainty into the system, which manifests as a non-zero conditional entropy between the two features. Hence, we can hypothesise that the samples diverging from the ground truth (indicated by the blue Xs) are either real, or are present due to the introduction of erroneous data points. This allows us to set up a hypothesis test, which we introduce in Radley et al. (2023) as the ES hypothesis test. The ES hypothesis test allows us to quantify whether it is more likely or not than random chance that the overlapping RF majority and QF minority states are present due to sampling/measurement error.

All five of these error scenarios are encoded into our cESFW algorithm, which can be inspected to gain further insights into the algorithmic application of the error scenarios. However, before looking at our cESFW code, we advise reading the relevant error scenario sections in the supplementary material of our Radley et al. (2023) publication for a detailed description.

### Continuous Entropy Sort Feature Weighting workflow

In Fig. 1, we outlined the main steps in our proposed cESFW scRNA-seq data processing workflow. In this section we will provide further details for each step. Executable code to apply our cESFW workflow to the synthetic and human embryo scRNA-seq data presented in this paper can be found at https://github.com/aradley/cESFW_Embryo_Topology_Paper. The primary purpose of this section and our workflow is to give readers an insight into how cESFW can be successfully applied to scRNA-seq data. It is expected that users will find other ways to incorporate cESFW into their omics data analysis pipelines for the plethora of data that is now available to the community.

#### Temporary minority state normalisation

For features to be compared with one another with cESFW, they must each be converted into their minority state feature activity vectors (Fig. S14A). Practically, converting features into their minority state vectors amounts to normalising each feature so that their values are within the same range, in a manner that meaningfully captures how features co-occur with one another. For gene expression data, this could be two genes where when one feature displays expression values close to its maximum expression in the data, the other gene also shows values close to its maximum expression. In this example, to convert these features to their minority state feature activity vectors, we could simply divide all the values of each feature by their respective feature maximums (Fig. S14C). However, now consider that there is a third gene where, when the first two genes have values close to 0, the third gene has values close to its maximum expression, and when the first two features have values close to their maximum, the third feature has values close to 0 (Fig. S14A,B). This is equivalent to the high expression of the third gene being the majority state feature activity (Fig. S14B), which is implicitly defined by the sum of activities in the normalised vector of the third gene being greater than half the number of samples. Hence, to convert the third gene to its minority state activity vector, we simply deduct each value of the vector from 1 (Fig. S14A,B).

This logic is generally applicable to any dataset where we can justifiably convert each feature into active versus inactive states. How this abstraction is done and whether this is meaningful for different datasets is context dependent and ultimately left to the user. In this work, we found the relatively simple normalisation method presented in Table S2 is effective.

In this work, the feature percentile threshold (*p*) was set as the 97.5th percentile. Sensitivity analysis demonstrated that the results of downstream cESFW analysis were robust to different choices of *p* (Fig. S12). Having obtained the minority state matrix (*M_m_*), we may now use it as the input for ES calculations to quantify the correlations between features.

#### Exclude genes significantly enriched within a dataset [optional]

Sometimes when analysing scRNA-seq data we would like to combine datasets from different sequencing experiments. Combining datasets from different experiments can introduce batch effects as a significant confounder in differentiating biological signal from experimental noise. To mitigate the contribution of batch effects on downstream analysis of scRNA-seq data, several methods have been developed (Tran et al., 2020). Many of these methods focus on smoothing or regressing the counts matrices with the aim of making samples with similar cellular states that are derived from different datasets look more similar to one another than samples of different cellular states that are from the same dataset. Although there are scenarios where smoothing/imputation methods can be successfully employed to gain greater insights into scRNA-seq datasets, they always incur the risk of adding computational artefacts or unintentionally removing biological signal of interest.

In this cESFW workflow, we address the issue of batch effects in a different manner. Rather than change the values of the counts matrix, we hypothesised that genes that are significantly enriched in any individual dataset constitute genes that contribute to batch effects and hence should be removed from the data. In doing so, we aim to reduce batch effect signals to the point that biological patterns of interest that are common between datasets have a larger signal than dataset-specific noise, which should in turn lead to more biologically relevant insights in downstream analysis.

To identify which genes are considered significantly enriched in a specific dataset in an unsupervised manner, we turn to the Error Potential (EP). The EP is a metric derived from ES and described in detail by Radley et al. (2023). Given two minority state activity feature vectors, the EP between the two features can be calculated, and if the EP>0, the features can be considered to be co-occurring with each other in a manner that is greater than random chance. In Table S3 we provide pseudocode for how we use the EP for excluding features that are significantly enriched in any of the datasets comprising a scRNA-seq counts matrix of interest.

A limitation with this batch-specific gene exclusion methodology is that, if there are datasets within the combined counts matrix (*M*) that are comprised of almost entirely one cell type, and that cell type has a distinct gene signature that is not present in any other dataset, these genes may be unwittingly identified as batch effect genes and removed from the data. In such a scenario, it would be up to the researcher to modify the workflow accordingly or seek alternative methodologies.

#### Calculate ESS and EP feature matrices

Having created the *M_m_*, we use it to calculate the ESS and EP pairwise for each gene in *M_m_*. In doing so we generate the *ESSs* and *EPs* matrices, which are *j* by *j* matrices in which *j* is the number of genes present in *M_m_*. The ESS uses ES theory (Fig. S15) to quantify the correlation between two features with a value between −1 and 1. The EP uses a hypothesis test between the prospect of two features being completely independent versus the two features being dependent, to quantify the significance of the correlation between the two features. When EP<0, it is more likely that the observed correlation between two features occurred through a random relationship between two independent features than the features having a dependent relationship. Conversely, when EP>0, the correlation between the two features is more likely to have been observed due to some dependent relationship.

For further details regarding the theory and derivation behind the ESS and EP, see Radley et al. (2023). In the next steps of our cESFW workflow, we use the *ESSs* and *EPs* matrices to get a feature importance weight for each of the *j* genes, and use these weights to decide which genes should be retained in the scRNA-seq counts matrix, and which should be excluded.

#### Calculate cESFW feature weights

Having generated the *ESSs* and *EPs* matrices, we now calculate the cESFW feature weights for each of the *j* genes. To calculate gene weights, we combine the *ESSs* and *EPs* matrices derived from ES theory, with the concept of node centrality from graph theory. In the context of scRNA-seq data, node centrality can be used to try to distinguish between genes (nodes) that form networks with relatively high correlations with other genes in the dataset, from genes that form weak correlative networks. The assumption is that genes that are members of highly correlating networks are more likely to be involved in cellular function/identity.

Because the ESS is a symmetric correlation metric, the *ESSs* matrix can be thought of as a weighted undirected graph in the same manner as other correlation metrics (Pearson's correlation, mutual information, etc.), where *ESSs* [2, 4]=*ESSs* [4, 2]=0*.*7 would quantify the weight of the edge between genes 2 and 4 as 0.7. However, practical limitations of node centrality often force users to apply supervision to the analysis of complex datasets such as scRNA-seq data. For example, Singh et al. (2020) discuss how node centrality measures that use weighted edges can fail to account for the number of edges that each gene has to other genes. In the following we show that with the *ESSs_jx j_* and *EPs_jx j_* matrices we can calculate node centralities of weighted networks in a manner that is unsupervised and accounts for the number of edges that each gene has to other genes.

As stated, *ESSs* can be used as the weighted graph. Now note that each entry of *EPs* quantifies to what degree the corresponding correlation value in *ESSs_jx j_* is likely to have occurred by random chance or a dependent relationship between a pair of features. According to ES hypothesis testing (Radley et al., 2023), whenever EP<0, the feature pair correlation can be assumed to have occurred by random chance. We use this property to create a dependent *EPs* matrix, *dEPs*, where *dEPs* is created by setting all values in *EPs*<0 to 0. *dEPs* now quantifies all the pairwise gene relationships for which co-expression occurs to a degree greater than random chance. Now we can calculate the weighted node centrality of each gene as the weighted *j*th column averages of *ESSs* with *dEPs* as weights such that:
(1)

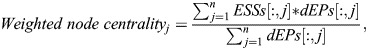
where *j* denotes the column/gene index in *ESSs* and *dEPs*. By weighting each value in *ESSs* by the corresponding values in *dEPs*, we mitigate the contribution of uninformative/random correlative patterns to the calculation of gene centrality.

Finally, we encode a biologically inspired assumption into the weighted node centrality *j* to get our final cESFW gene weights. Because every non-zero value in *dEPs* indicates that a pair of genes have a quantifiably significant relationship, we can count the number of quantifiably meaningful edges a gene has with other genes in the data. We then seek to penalise genes that have significantly more edges than others through the following:
(2)

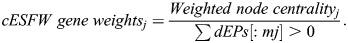
The biological assumption behind Eqn 2 is that cell identity and differentiation is controlled by relatively small networks of tightly controlled genes. Hence, genes with relatively high weighted node centralities (Eqn 1) and relatively low numbers of significant gene co-regulation edges should be more informative of cellular identity. Conversely, genes that have relatively low weighted node centralities and/or relatively high numbers of significant gene co-regulation edges should constitute genes that are weakly or non-informative of cell identity.

In summary, we have calculated a vector of cESFW gene weights, for each gene in *ESSs_jx j_*, such that a larger cESFW gene weight indicates that the expression of a gene in the original scRNA-seq counts matrix are more likely to be part of a small network of highly correlated genes than other genes with lower cESFW gene weights. As such, we can use the cESFW gene weights to form a ranked list of genes for downstream analysis.

#### Select gene cluster with branching network

The final step of our proposed cESFW workflow is to take the cESFW gene weights and use them to select the top most informative genes for downstream analysis. In a similar manner to HVG analysis, we can simply take a subset of the top highest ranking genes according to the cESFW gene weights. Up until this point, the cESFW workflow has been entirely unsupervised, i.e., no subjective user decisions were applied and no prior knowledge regarding cell type or gene importance was used when deciding which genes to exclude from downstream analysis. As with HVG analysis, the final step of the cESFW workflow where we choose how many genes to select based on the cESFW gene weights is an iterative process where researchers use domain knowledge to settle on the final gene set, based on the assumption that the cESFW ranked gene list provides a meaningful proxy for thresholding. Below, we provide a description of our rationale for using the cESFW gene weights to identify a set of biologically informative genes for the human embryo scRNA-seq data within this manuscript. We present this description as a guide to aid analysis on other datasets.

For the human embryo scRNA-seq data presented in this manuscript, we selected the top 4000 genes by taking the 4000 genes with the highest cESFW gene weights. The selection threshold of 4000 was chosen by looking at UMAPs of the genes in the *ESSs* matrix at varying cESFW gene weight cutoffs (Fig. S13A). In these UMAPs, we qualitatively look for two main properties. First, we are looking for clusters of genes that have significant branching patterns, rather than smooth ‘blob’ like clusters. In Fig. S13A we see that as we decrease the number of top ranked genes that we include, the darker blue coloured cluster forms increasingly distinct branches. These branches indicate that there are genes that form fully connected networks, with some genes being very specifically connected to each other without being directly connected to many other genes in the network. Biologically we infer that these represent genes that are specifically expressed in a subset of cells present in the entire dataset, and are hence genes of interest when dissecting cellular identity. The top 4000 genes were chosen relatively arbitrarily as the point at which a clear branching pattern appears in the dark blue cluster. Readers will notice that choosing values smaller than 4000 can result in even more distinct branches in the blue cluster. We suggest users of our cESFW workflow aim to find a balance between keeping as many genes as possible while increasing the qualitative property of branching structure. Furthermore, we suggest supervising the selection of gene clusters using a small set of markers known to be important in the system of study. In this work, we found that genes known to be important during early human embryo development (Fig. S4) are enriched in the dark blue cluster of genes, further suggesting that this cluster of genes is more likely to discriminate cell type identities in downstream analysis.

A second feature of interest in these UMAPs is that a separate cluster of genes with significantly higher weighted node centrality scores can appear (yellow; Fig. S13A). Empirically, we find that failure to remove these clusters of genes prevents the emergence of high resolution scRNA-seq embeddings (see the online GitHub workflow for examples). Gene set enrichment analysis of the final 3012 gene set used throughout this manuscript, comprising the blue cluster of the top 4000 genes in Fig. S13A (red square), showed that these genes were enriched for GO terms relating to regulation and development (Fig. S13B). Conversely, the GO terms of the 988 genes comprising the yellow cluster were dominated by terms relating to transcription, translation and metabolism (Fig. S13C). We therefore hypothesise that the genes present in the relatively highly correlated yellow clusters represent expression signals relating to generic cell transcription, translation and metabolism processes which are less discriminating of cellular identity/function than genes that are part of the blue branching cluster. By excluding the genes from the yellow cluster in downstream analysis, we appear to further increase the signal to noise ratio of cell-type-specific expression, which ultimately improves our ability to discriminate different cell identities in the data.

We emphasise that this final step of the cESFW workflow is supervised and subject to user interpretation/dataset variability. We hope that our transparency regarding this step helps guide potential users of our cESFW workflow to improved resolution in their datasets or to develop new cESFW feature weight based selection criteria.

### Data and code availability

Instructions to install cESFW can be found at: https://github.com/aradley/cESFW. Computational workflows and data used for the generation of results in this article can be found at: https://github.com/aradley/cESFW_Embryo_Topology_Paper. A copy of the human pre- and post-implantation embryo data used in this work may be found in the following permanent Mendeley Data repository: https://doi.org/10.17632/34td4ds2r9.1.

The data used to create the UMAP embedding are a combination of human embryo pre-implantation and post-implantation embryo (extended culture) scRNA-seq data. The pre-implantation raw counts scRNA-seq data from Yan et al. (2013), Petropoulos et al. (2016), Fogarty et al. (2017) and Meistermann et al. (2021) were compiled into a single gene expression matrix by Meistermann et al. (2021). For information regarding quality control and cell filtering of these four datasets, please refer to Meistermann et al. (2021). The Yanagida et al. (2021) human pre-implantation embryo data are available via GEO accession number GSE171820. The 3D cultured post-implantation human embryo raw counts scRNA-seq data are from Xiang et al. (2020), available at GSE136447. For our analysis, we removed the day 6 and 7 cultured embryo samples from Xiang et al. (2020) to mitigate the contribution of batch effects and uncertainty over timings of embryo stages.

### Synthetic data generation

The four SDs used in this study were generated using the Dyngen software (Cannoodt et al., 2021). Synthetic dataset size and shapes were all controlled using out-of-box Dyngen functions.

### UMAPs

For consistency, all UMAPs presented in this work were created with the Python umap-learn package, with n_neighbors and min_dist set to 50 and 0.1 respectively. The correlation distance metric was used instead of the default Euclidean distance metric. All UMAPs displaying gene expression profiles show the *log*_2_ normalised gene expression. No data smoothing or clipping were applied. An interactive online portal where readers may visualise gene expression profiles on our UMAP embedding and generate their own plots can be found at: https://bioinformatics.crick.ac.uk/shiny/users/boeings/radley2024umap_app/.

### Unsupervised clustering

Unsupervised clustering of scRNA-seq embryo data (Fig. S3A) was performed using the Python package sklearn.cluster.AgglomerativeClustering, with *k*=30 clusters and the correlation distance metric.

### Gene marker identification

Identification of markers for UMAP plotting and our ranked gene list (Table S1) was performed by using the ESS correlation metric to rank genes that are enriched to a cell state or cell states of interest. For details see Radley et al. (2023), and for examples see the workflows provided alongside this paper (https://github.com/aradley/cESFW_Embryo_Topology_Paper).

### RNA velocity

RNA velocity was carried out using the scVelo (Bergen et al., 2020) with default parameters, except for the cell nearest neighbour matrix, which we manually substituted with the cell nearest neighbour matrix according to the 3012 cESFW genes and the correlation distance metric.

### Query cell classification

Query scRNA-seq samples were transformed onto our UMAP embedding using the out-of-box umap.transform function. After query samples are placed on the UMAP embedding, we may predict their cell type in the low dimensional UMAP space.

To predict cell identity, we used the sklearn.neighbors.KNeighborsClassifier Python function. A knn classifier was trained using our UMAP embedding and cell state annotations. The number of neighbours used to train the classifier was 20. The trained classifier was then applied to the query samples to get their predicted cell identities.

### Lineage emergence during blastocyst formation

To identify the emergence of Epi, Hyp and TE gene expression profiles during human blastocyst formation, we used the ESS to rank genes by how well their expression profiles overlap with the morula to Epi, Hyp or TE trajectories, and then progressively restricted the late blastocyst Epi, Hyp or TE populations. As an example, to identify Epi staging genes, we started by ranking genes based on their enrichment in the combined morula, ICM/TE branch, ICM and preIm-epi samples from our cell UMAP annotations (Fig. 3C). This was followed by ranking against the combined ICM/TE branch, ICM and preIm-Epi population, and then by the combined ICM and preIm-Epi samples, and finally the preIm-Epi samples in isolation (Fig. S10A). A similar process was repeated for the Hyp and TE trajectories. For each ranked gene list generated, we inspected the top 30 ranked genes and manually confirmed the gene expression profile using the UMAP embedding. Through this process, we were able to identify sets of genes that delineate the emergence of Epi, Hyp and TE lineages from the morula progenitor population.

### Individual embryo cell state co-occurrence

As the ESS is a correlation metric that quantifies the co-occurrence of states in a set of samples, we used it to quantify how often our cell state annotations coincided in individual embryos. By treating each embryo as a sample and counting how many times each cell label appears for each embryo, we create a sample by feature matrix. We normalise each sample by dividing all values by the total number of cells in each embryo, and then calculate the ESS from the resulting matrix. This generates the ESS correlation matrix, presented in Fig. 4A,B.

## Supplementary Material



10.1242/develop.202832_sup1 Supplementary information

Table S1 Human embryo scRNA-seq cell type ranked gene lists. Ranked gene lists created using the Entropy Sort Score (ESS) correlation metric to rank the enrichment of each of the 16719 genes in each of the 15 annotated cell types.

## References

[DEV202832C1] Allègre, N., Chauveau, S., Dennis, C., Renaud, Y., Meistermann, D., Estrella, L. V., Pouchin, P., Cohen-Tannoudji, M., David, L. and Chazaud, C. (2022). NANOG initiates epiblast fate through the coordination of pluripotency genes expression. *Nat. Commun.* 13, 3550. 10.1038/s41467-022-30858-8 35729116 PMC9213552

[DEV202832C2] Andrews, T. S. and Hemberg, M. (2018). False signals induced by single-cell imputation. *F1000Research* 7, 1740. 10.12688/f1000research.16613.1 30906525 PMC6415334

[DEV202832C3] Bellman, R. (1957). Dynamic programming. *Science* 153, 34-37. 10.1126/science.153.3731.34 17730601

[DEV202832C4] Bergen, V., Lange, M., Peidli, S., Wolf, F. A. and Theis, F. J. (2020). Generalizing RNA velocity to transient cell states through dynamical modeling. *Nat. Biotechnol.* 38, 1408-1414. 10.1038/s41587-020-0591-3 32747759

[DEV202832C5] Cannoodt, R., Saelens, W., Deconinck, L. and Saeys, Y. (2021). Spearheading future omics analyses using dyngen, a multi-modal simulator of single cells. *Nat. Commun.* 12, 3942. 10.1038/s41467-021-24152-2 34168133 PMC8225657

[DEV202832C6] Chen, H., Albergante, L., Hsu, J. Y., Lareau, C. A., Lo Bosco, G., Guan, J., Zhou, S., Gorban, A. N., Bauer, D. E., Aryee, M. J. et al. (2019). Single-cell trajectories reconstruction, exploration and mapping of omics data with STREAM. *Nat. Commun.* 10, 1-14. 10.1038/s41467-019-09670-4 31015418 PMC6478907

[DEV202832C7] Chu, S.-K., Zhao, S., Shyr, Y. and Liu, Q. (2022). Comprehensive evaluation of noise reduction methods for single-cell RNA sequencing data. *Brief. Bioinform.* 23, bbab565. 10.1093/bib/bbab565 35048125 PMC8921632

[DEV202832C8] Cockburn, K. and Rossant, J. (2010). Making the blastocyst: lessons from the mouse. *J. Clin. Invest.* 120, 995-1003. 10.1172/JCI41229 20364097 PMC2846056

[DEV202832C9] Corujo-Simon, E., Radley, A. H. and Nichols, J. (2023). Evidence implicating sequential commitment of the founder lineages in the human blastocyst by order of hypoblast gene activation. *Development* 150, dev201522. 10.1242/dev.201522 37102672 PMC10233721

[DEV202832C10] Dattani, A., Corujo-Simon, E., Radley, A., Heydari, T., Taheriabkenar, Y., Carlisle, F., Lin, S., Liddle, C., Mill, J., Zandstra, P. et al. (2024). Naive pluripotent stem cell-based models capture FGF-dependent human hypoblast lineage specification. *Cell Stem Cell*, 31, 1-14. 10.1016/j.stem.2024.05.003 38823388

[DEV202832C11] David, L., Bruneau, A., Fréour, T., Rivron, N. and Van de Velde, H. (2023). An update on human pre- and peri-implantation development: a blueprint for blastoids. *Curr. Opin. Genet. Dev.* 83, 102125. 10.1016/j.gde.2023.102125 37801801

[DEV202832C12] Fogarty, N. M., McCarthy, A., Snijders, K. E., Powell, B. E., Kubikova, N., Blakeley, P., Lea, R., Elder, K., Wamaitha, S. E., Kim, D. et al. (2017). Genome editing reveals a role for OCT4 in human embryogenesis. *Nature* 550, 67-73. 10.1038/nature24033 28953884 PMC5815497

[DEV202832C13] Gao, X., Nowak-Imialek, M., Chen, X., Chen, D., Herrmann, D., Ruan, D., Chen, A. C. H., Eckersley-Maslin, M. A., Ahmad, S., Lee, Y. L. et al. (2019). Establishment of porcine and human expanded potential stem cells. *Nat. Cell Biol.* 21, 687-699. 10.1038/s41556-019-0333-2 31160711 PMC7035105

[DEV202832C14] Goedel, A. and Lanner, F. (2021). A peek into the black box of human embryology. *Nature* 600, 223-224. 10.1038/d41586-021-03381-x 34789887

[DEV202832C15] Grau, J., Grosse, I. and Keilwagen, J. (2015). PRROC: computing and visualizing precision-recall and receiver operating characteristic curves in R. *Bioinformatics* 31, 2595-2597. 10.1093/bioinformatics/btv153 25810428 PMC4514923

[DEV202832C16] Guo, G., von Meyenn, F., Rostovskaya, M., Clarke, J., Dietmann, S., Baker, D., Sahakyan, A., Myers, S., Bertone, P., Reik, W. et al. (2017). Epigenetic resetting of human pluripotency. *Development* 144, 2748-2763. 10.1242/dev.146811 28765214 PMC5560041

[DEV202832C17] Guo, G., Stirparo, G. G., Strawbridge, S. E., Spindlow, D., Yang, J., Clarke, J., Dattani, A., Yanagida, A., Li, M. A., Myers, S. et al. (2021). Human naive epiblast cells possess unrestricted lineage potential. *Cell Stem Cell* 28, 1040-1056.e6. 10.1016/j.stem.2021.02.025 33831366 PMC8189439

[DEV202832C18] Haque, A., Engel, J., Teichmann, S. A. and Lönnberg, T. (2017). A practical guide to single-cell RNA-sequencing for biomedical research and clinical applications. *Genome Med.* 9, 1-12. 10.1186/s13073-017-0467-4 28821273 PMC5561556

[DEV202832C19] Heumos, L., Schaar, A. C., Lance, C., Litinetskaya, A., Drost, F., Zappia, L., Lücken, M. D., Strobl, D. C., Henao, J., Curion, F. et al. (2023). Best practices for single-cell analysis across modalities. *Nat. Rev. Genet.* 24, 550-572. 10.1038/s41576-023-00586-w 37002403 PMC10066026

[DEV202832C20] Liu, D., Chen, Y., Ren, Y., Yuan, P., Wang, N., Liu, Q., Yang, C., Yan, Z., Yang, M., Wang, J. et al. (2022). Primary specification of blastocyst trophectoderm by scRNA-seq: new insights into embryo implantation. *Sci. Adv.* 8, 3725. 10.1126/sciadv.abj3725 PMC9365277 35947672

[DEV202832C21] Mazid, M. A., Ward, C., Luo, Z., Liu, C., Li, Y., Lai, Y., Wu, L., Li, J., Jia, W., Jiang, Y. et al. (2022). Rolling back human pluripotent stem cells to an eight-cell embryo-like stage. *Nature* 605, 315-324. 10.1038/s41586-022-04625-0 35314832

[DEV202832C22] McInnes, L., Healy, J., Saul, N. and Großberger, L. (2018). UMAP: uniform manifold approximation and projection. *J. Open Source Softw.* 3, 861. 10.21105/joss.00861

[DEV202832C23] Meistermann, D., Bruneau, A., Loubersac, S., Reignier, A., Firmin, J., François-Campion, V., Kilens, S., Lelièvre, Y., Lammers, J., Feyeux, M. et al. (2021). Integrated pseudotime analysis of human pre-implantation embryo single-cell transcriptomes reveals the dynamics of lineage specification. *Cell Stem Cell* 28, 1625-1640.e6. 10.1016/j.stem.2021.04.027 34004179

[DEV202832C24] Messmer, T., von Meyenn, F., Savino, A., Santos, F., Mohammed, H., Lun, A. T. L., Marioni, J. C. and Reik, W. (2019). Transcriptional heterogeneity in naive and primed human pluripotent stem cells at single-cell resolution. *Cell Rep.* 26, 815-824.e4. 10.1016/j.celrep.2018.12.099 30673604 PMC6344340

[DEV202832C25] Nakamura, T., Yabuta, Y., Okamoto, I., Sasaki, K., Iwatani, C., Tsuchiya, H. and Saitou, M. (2017). Single-cell transcriptome of early embryos and cultured embryonic stem cells of cynomolgus monkeys. *Sci. Data* 4, 170067. 10.1038/sdata.2017.67 28649393 PMC5477564

[DEV202832C26] Nichols, J. and Smith, A. (2009). Naive and primed pluripotent states. *Cell Stem Cell* 4, 487-492. 10.1016/j.stem.2009.05.015 19497275

[DEV202832C27] Petropoulos, S., Edsgärd, D., Reinius, B., Deng, Q., Panula, S. P., Codeluppi, S., Plaza Reyes, A., Linnarsson, S., Sandberg, R. and Lanner, F. (2016). Single-cell RNA-seq reveals lineage and X chromosome dynamics in human preimplantation embryos. *Cell* 165, 1012-1026. 10.1016/j.cell.2016.03.023 27062923 PMC4868821

[DEV202832C28] Posfai, E., Petropoulos, S., de Barros, F. R. O., Schell, J. P., Jurisica, I., Sandberg, R., Lanner, F. and Rossant, J. (2017). Position- and Hippo signaling-dependent plasticity during lineage segregation in the early mouse embryo. *eLife* 6, e22906. 10.7554/eLife.22906 28226240 PMC5370188

[DEV202832C29] Radley, A., Corujo-Simon, E., Nichols, J., Smith, A. and Dunn, S. J. (2023). Entropy sorting of single-cell RNA sequencing data reveals the inner cell mass in the human pre-implantation embryo. *Stem Cell Rep.* 18, 47-63. 10.1016/j.stemcr.2022.09.007 PMC9859930 36240776

[DEV202832C30] Singh, A., Singh, R. R. and Iyengar, S. R. S. (2020). Node-weighted centrality: a new way of centrality hybridization. *Comput. Social Networks* 7, 6. 10.1186/s40649-020-00081-w

[DEV202832C31] Singh, M., Kondrashkina, A. M., Widmann, T. J., Cortes, J. L., Bansal, V., Wang, J., Römer, C., Garcia-Canadas, M., Garcia-Perez, J. L., Hurst, L. D. et al. (2023). A new human embryonic cell type associated with activity of young transposable elements allows definition of the inner cell mass. *PLoS Biol.* 21, e3002162. 10.1371/journal.pbio.3002162 37339119 PMC10281584

[DEV202832C32] Smith, A. (2017). Formative pluripotency: the executive phase in a developmental continuum. *Development* 144, 365-373. 10.1242/dev.142679 28143843 PMC5430734

[DEV202832C33] Stirparo, G. G., Boroviak, T., Guo, G., Nichols, J., Smith, A. and Bertone, P. (2018). Integrated analysis of single-cell embryo data yields a unified transcriptome signature for the human pre-implantation epiblast. *Development (Camb.)* 145, dev158501. 10.1242/dev.158501 PMC5818005 29361568

[DEV202832C34] Stuart, T., Butler, A., Hoffman, P., Hafemeister, C., Papalexi, E., Mauck, W. M., Hao, Y., Stoeckius, M., Smibert, P. and Satija, R. (2019). Comprehensive integration of single-cell data. *Cell* 177, 1888-1902.e21. 10.1016/j.cell.2019.05.031 31178118 PMC6687398

[DEV202832C35] Takashima, Y., Guo, G., Loos, R., Nichols, J., Ficz, G., Krueger, F., Oxley, D., Santos, F., Clarke, J., Mansfield, W. et al. (2014). Resetting transcription factor control circuitry toward ground-state pluripotency in human. *Cell* 158, 1254-1269. 10.1016/j.cell.2014.08.029 25215486 PMC4162745

[DEV202832C36] Tang, F., Barbacioru, C., Wang, Y., Nordman, E., Lee, C., Xu, N., Wang, X., Bodeau, J., Tuch, B. B., Siddiqui, A. et al. (2009). mRNA-Seq whole-transcriptome analysis of a single cell. *Nat. Methods* 6, 377-382. 10.1038/nmeth.1315 19349980

[DEV202832C37] Taubenschmid-Stowers, J., Rostovskaya, M., Santos, F., Ljung, S., Argelaguet, R., Krueger, F., Nichols, J. and Reik, W. (2022). 8C-like cells capture the human zygotic genome activation program in vitro. *Cell Stem Cell* 29, 449. 10.1016/j.stem.2022.01.014 35216671 PMC8901440

[DEV202832C38] Tran, H. T. N., Ang, K. S., Chevrier, M., Zhang, X., Lee, N. Y. S., Goh, M. and Chen, J. (2020). A benchmark of batch-effect correction methods for single-cell RNA sequencing data. *Genome Biol.* 21, 12. 10.1186/s13059-019-1850-9 31948481 PMC6964114

[DEV202832C39] Wei, X., Fang, X., Yu, X., Li, H., Guo, Y., Qi, Y., Sun, C., Han, D., Liu, X., Li, N. et al. (2023). Integrative analysis of single-cell embryo data reveals transcriptome signatures for the human pre-implantation inner cell mass. *Dev. Biol.* 502, 39-49. 10.1016/j.ydbio.2023.07.004 37437860

[DEV202832C40] Xiang, L., Yin, Y., Zheng, Y., Ma, Y., Li, Y., Zhao, Z., Guo, J., Ai, Z., Niu, Y., Duan, K. et al. (2020). A developmental landscape of 3D-cultured human pre-gastrulation embryos. *Nature* 577, 537-542. 10.1038/s41586-019-1875-y 31830756

[DEV202832C41] Yabe, S., Alexenko, A. P., Amita, M., Yang, Y., Schust, D. J., Sadovsky, Y., Ezashi, T. and Roberts, R. M. (2016). Comparison of syncytiotrophoblast generated from human embryonic stem cells and from term placentas. *Proc. Natl. Acad. Sci. USA* 113, E2598-E2607. 10.1073/pnas.1601630113 27051068 PMC4868474

[DEV202832C42] Yan, L., Yang, M., Guo, H., Yang, L., Wu, J., Li, R., Liu, P., Lian, Y., Zheng, X., Yan, J. et al. (2013). Single-cell RNA-Seq profiling of human preimplantation embryos and embryonic stem cells. *Nat. Struct. Mol. Biol.* 20, 1131-1139. 10.1038/nsmb.2660 23934149

[DEV202832C43] Yanagida, A., Spindlow, D., Nichols, J., Dattani, A., Smith, A. and Guo, G. (2021). Naive stem cell blastocyst model captures human embryo lineage segregation. *Cell Stem Cell* 28, 1016-1022.e4. 10.1016/j.stem.2021.04.031 33957081 PMC8189436

[DEV202832C44] Yang, R., Goedel, A., Kang, Y., Si, C., Chu, C., Zheng, Y., Chen, Z., Gruber, P. J., Xiao, Y., Zhou, C. et al. (2021). Amnion signals are essential for mesoderm formation in primates. *Nat. Commun.* 12, 5126. 10.1038/s41467-021-25186-2 34446705 PMC8390679

[DEV202832C45] Yip, S. H., Sham, P. C. and Wang, J. (2019). Evaluation of tools for highly variable gene discovery from single-cell RNA-seq data. *Brief. Bioinform.* 20, 1583. 10.1093/bib/bby011 29481632 PMC6781572

[DEV202832C46] Yue, C., Chen, A. C. H., Tian, S., Fong, S. W., Lee, K. C., Zhang, J., Ng, E. H. Y., Lee, K. F., Yeung, W. S. B. and Lee, Y. L. (2020). Human embryonic stem cell-derived blastocyst-like spheroids resemble human trophectoderm during early implantation process. *Fertil. Steril.* 114, 653-664. 10.1016/j.fertnstert.2020.01.009 32444068

[DEV202832C47] Zadora, J., Singh, M., Herse, F., Przybyl, L., Haase, N., Golic, M., Yung, H. W., Huppertz, B., Cartwright, J. E., Whitley, G. et al. (2017). Disturbed placental imprinting in preeclampsia leads to altered expression of DLX5, a human-specific early trophoblast marker. *Circulation* 136, 1824-1839. 10.1161/CIRCULATIONAHA.117.028110 28904069 PMC5671803

