# Supplementary material for: Branching topology of the human embryo transcriptome revealed by Entropy Sort Feature Weighting
Source: Development. 2024 Jun 13;151(11):dev202832. doi: 10.1242/dev.202832 (PMC11213519; doi:10.1242/dev.202832)
Supplement: Supplementary information [file develop-151-202832-s1.pdf]

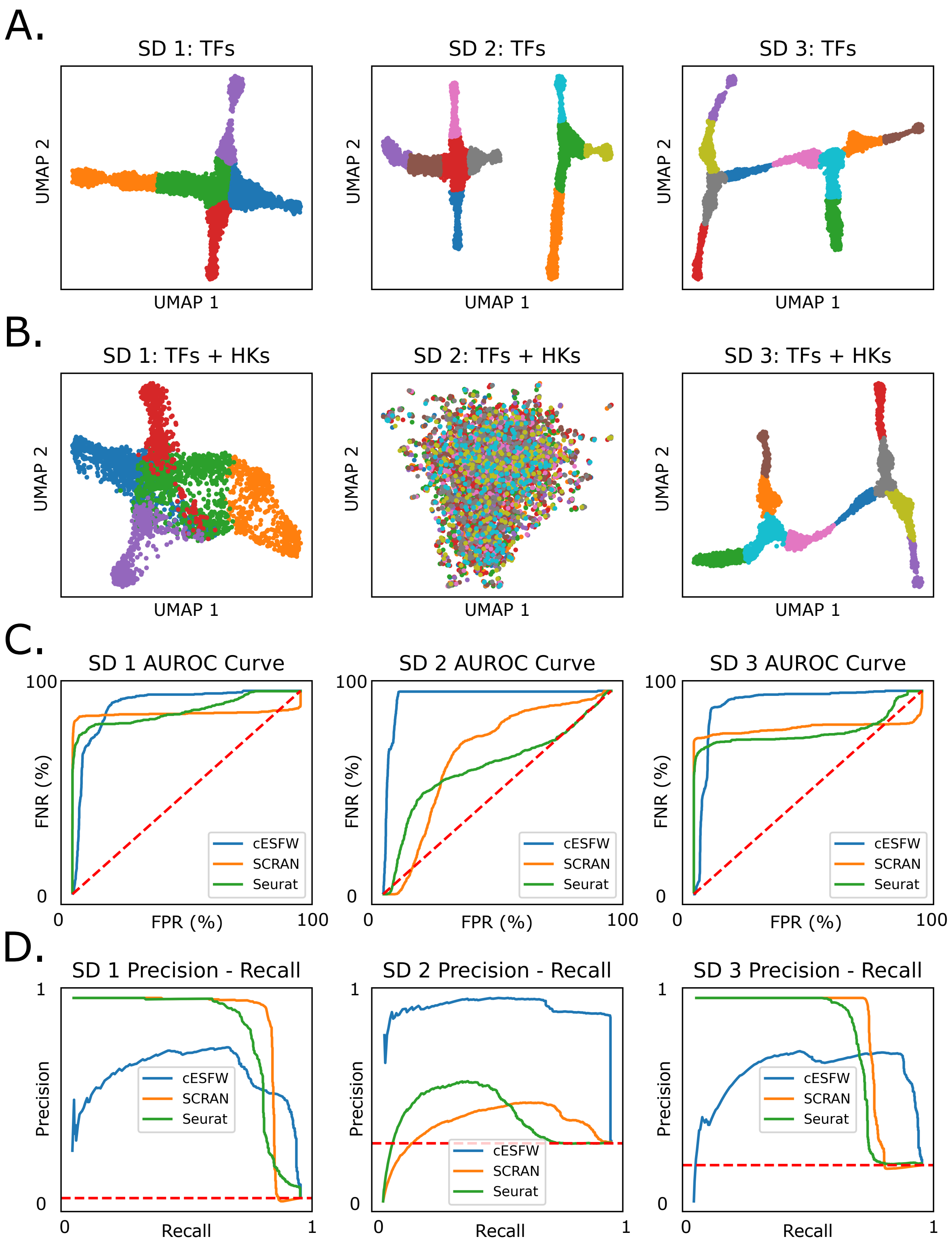

Fig. S1. Dyngen generated synthetic datasets (SDs) 1-3 A. UMAPs of SDs generated using just the TFs of each dataset. **B.** UMAPs of SDs generated using all of the TF and HK genes of each dataset. **C.** AUROC curves generated by different feature selection methods on each of SDs 1-3. **D.** PR-AUCs generated by different feature selection methods on each of SDs 1-3.

Development • Supplementary information

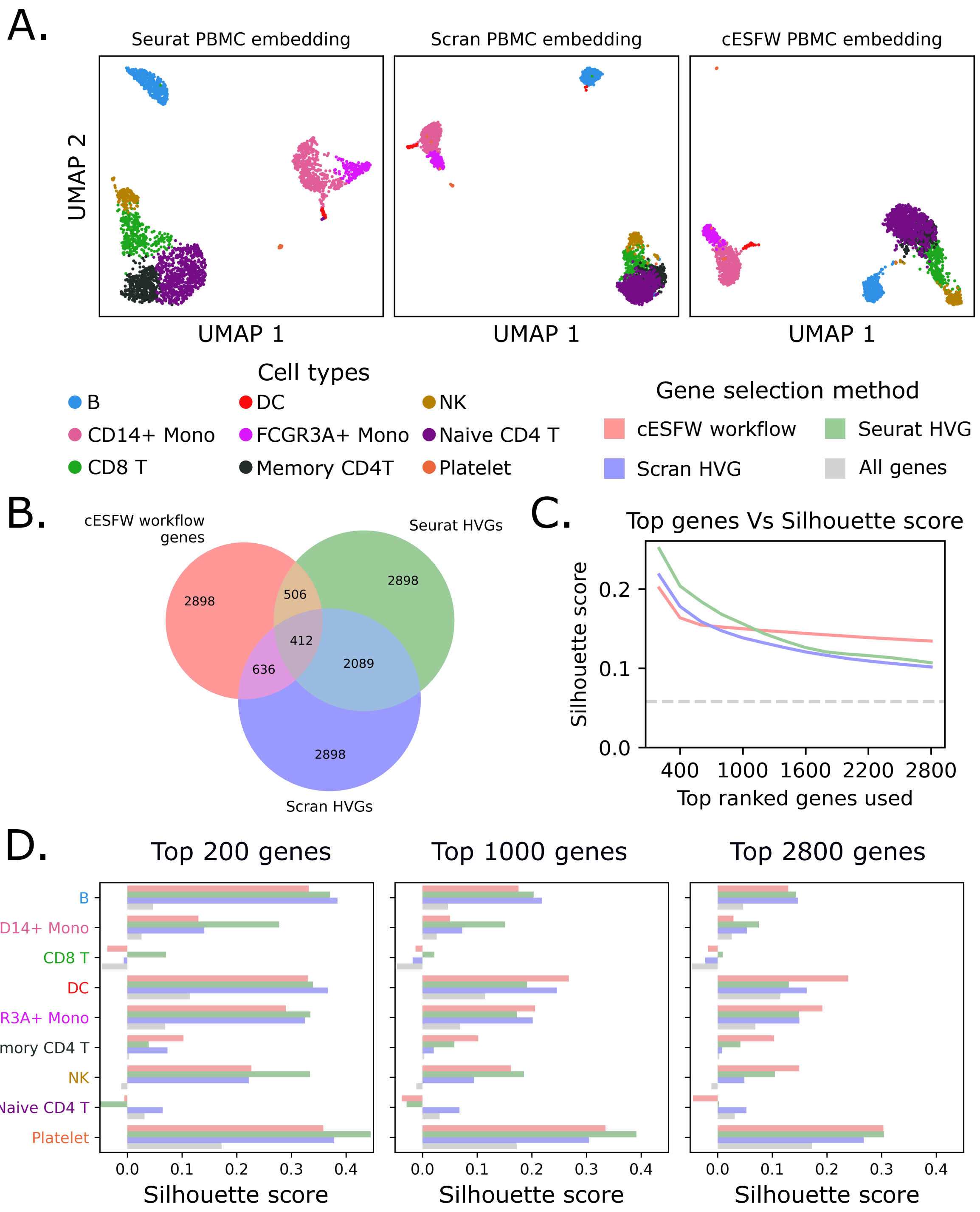

Fig. S2. Comparison of feature selection methods on Peripheral Blood Mononuclear Cells (PBMCs). We took PMBC scRNA-seq data from the Seurat tutorial workflow and performed feature selection with Seurat HVG selection, Scran HVG selection and our cESFW feature selection workflow. Cell type labels were defined through Seurat clustering and literature based marker genes. Using our cESFW workflow we identified a set of 2898 highly informative genes. **A.** Resulting UMAPs for each gene selection workflow. **B.** Venn diagram of the top 2898 genes from the cESFW, Seurat and Scran workflows. **C.** Average silhouette scores calculated at with varying sets of top ranked genes show that all three methods perform comparably. Grey dashed line shows the average silhouette score when using all genes in initial scRNA-seq counts matrix as a negative control. Although the cESFW initially performs slightly worse than Seurat and Scran, as more of the highest ranked genes are included, cESFW appears more stable. This is consistent with our results on synthetic data (Fig 2), where cESFW appears more robust to the presence of house keeping genes. **D.** Silhouette scores for individual clusters when using the top 200, 1000 or 2800 genes. As in (C.), we find that cESFW is initially comparable to Seurat and Scran HVG selection, but more stably maintains cell type clusters as the number of top ranked genes used increases.

Development • Supplementary information

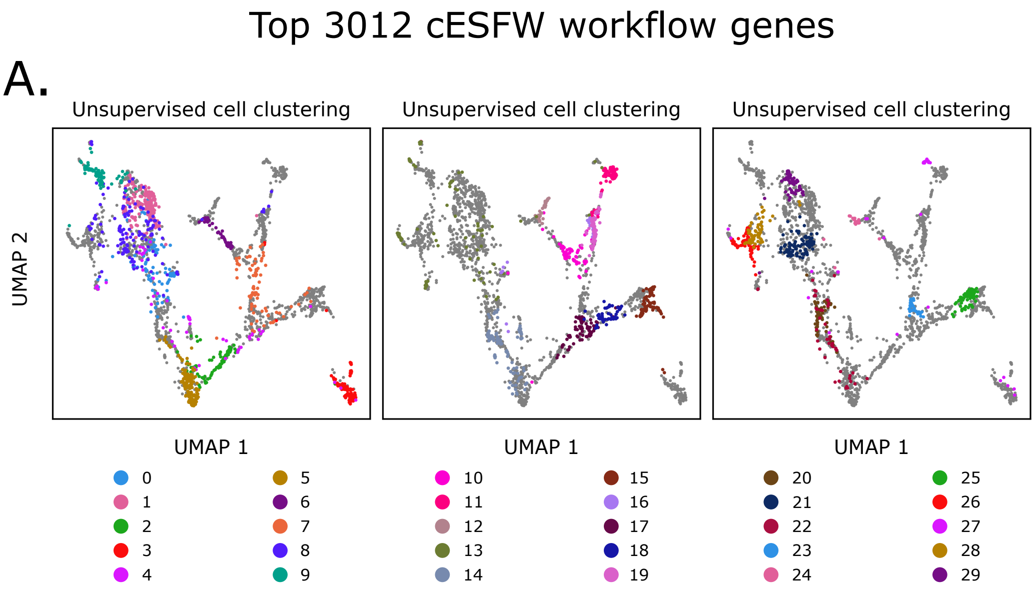

B.

Cell annotation	Unsupervised clusters	Number of cells	Cell annotation	Unsupervised clusters	Number of cells
8-cell	3	119	Hypo	6, 12, 24	111
Morula	15, 25	168	Early TE	2	91
ICM/TE branch	17, 18	136	Mid TE	0, 5, 14, 20, 22	470
ICM	7, 23	116	Mural TE	8, 21	235
Epi/Hypo branch	10	74	cTB	26, 28	128
preIm-Epi	16, 19	94	Polar TE	1, 29	261
Embryonic disc	11	95	sTB	9	96
ExE-Mech	27	37	Uknown	4, 13	169

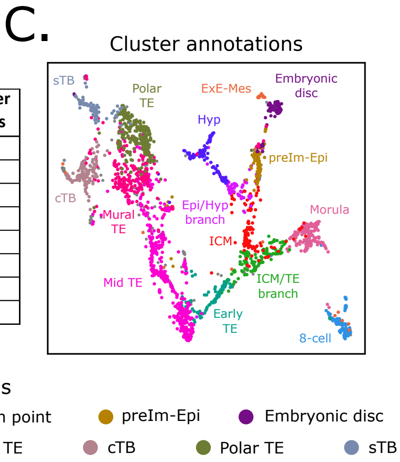

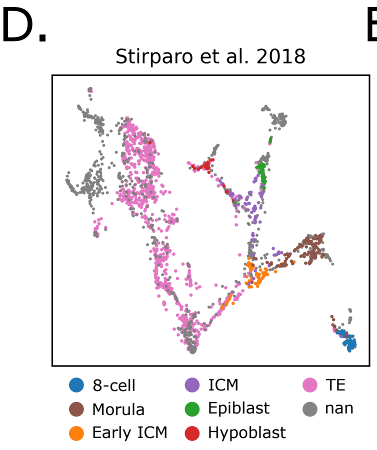

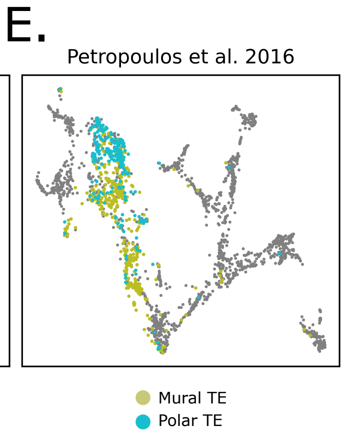

Fig. S3. Annotation of our UMAP embedding. Unsupervised agglomerative clustering with number of clusters set to 30. Clustering was performed on the raw counts matrix subsetted down to our 3012 cESFW selected genes. **A.** The resulting sample clusters visualised on our UMAP embedding. By inspecting known markers and previous analyses of these scRNA-seq data, we manually groups our unsupervised clusters into annotated cell types. **B.** Summary of the annotated cell types and which unsupervised clusters were used to form them. **C.** Visualisation our our annotated cell types on our UMAP embedding. **D.** Stirparo et al. 2018's supervised analysis of the Petropoulos et al. 2016 scRNA-seq data support our cell type annotations. **E.** Petropoulos et al. 2016's supervised analysis for differentiating between mural and polar TE support our cell type annotations.

Development • Supplementary information

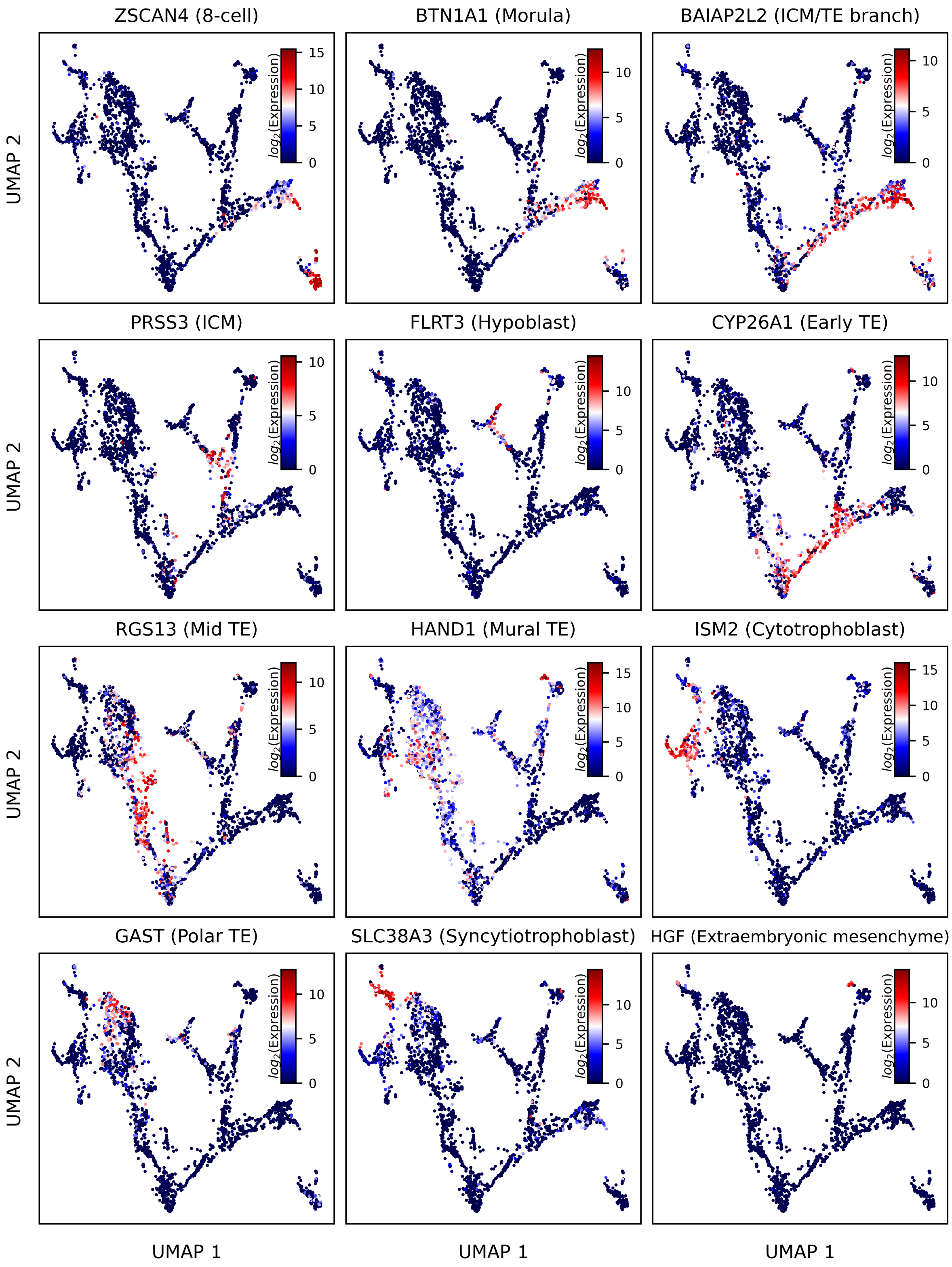

Fig. S4. Visualisation of cell type markers supported by the literature. Marker gene references are as follow: **8-cell** - Taubenschmid-Stowers et al. 2022; **Morula** - Stirparo et al. 2018; **ICM/TE branch** - Singh et al. 2023; **ICM** - A. Radley et al. 2023; **Hypoblast** - Corujo-Simon, A. H. Radley, and Nichols 2023; **Early TE** - Liu et al. 2022; **Mid TE** - Zadora et al. 2017; **Mural TE** - Liu et al. 2022; **Cytotrophoblast** - Li, Kurosawa, and Iwata 2019; **Polar TE** - Yue et al. 2020; **Syncytiotrophoblast** - Yabe et al. 2016; **Extraembryonic mesenchyme** - Yang et al. 2021.

Development • Supplementary information

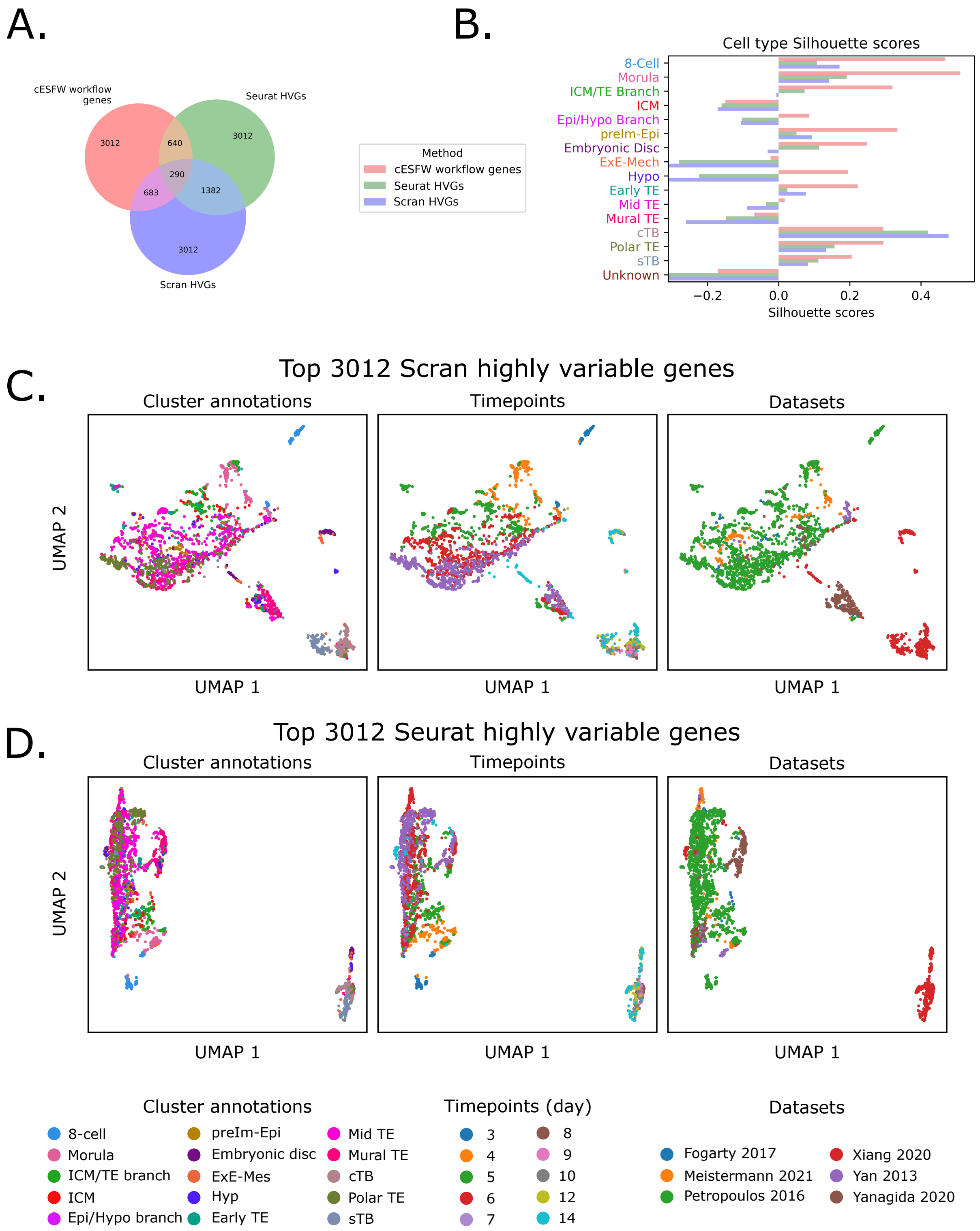

Fig. S5. Comparison 3012 cESFW workflow genes against top 3012 Scran and Seurat HVGs. A. Venn diagram of overlap between the genes within each of the 3012 gene sets. **B.** Silhouette scores of each of our annotated clusters when using each of the 3012 gene sets. **B, C.** UMAP generated when using the same scRNA-seq counts matrix and UMAP parameters used to generate our cESFW UMAP embedding, but instead using the top 3012 Scran HVGs (B.) or top 3012 Seurat HVGs (C.).

Development • Supplementary information

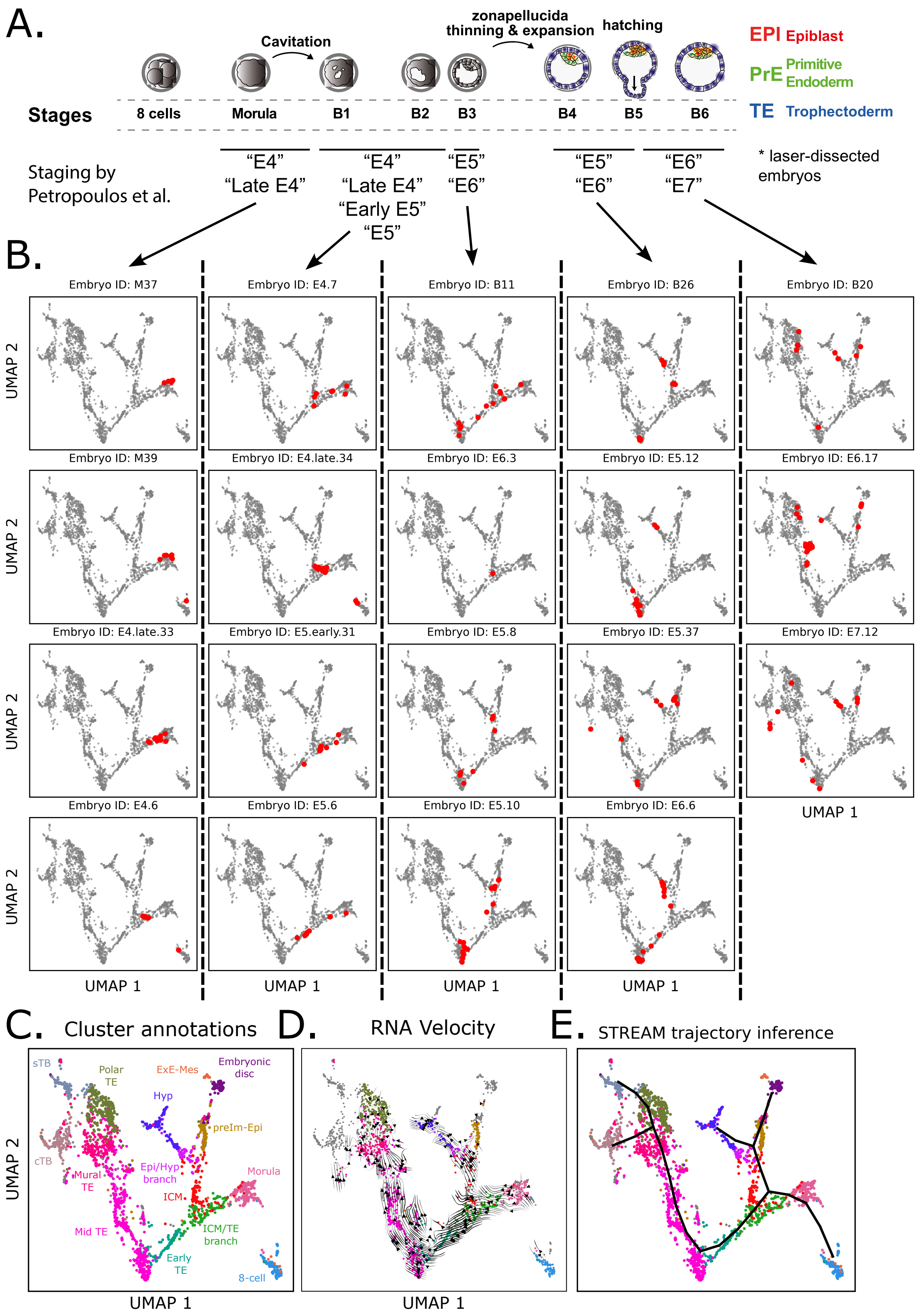

Fig. S6. Analysis of embryo cell state co-occurrence and differentiation trajectories. A, B. To support the results of the heatmaps in Fig 4D and E, we adapted Figure S2 from Meistermann et al. 2021 with the authors permission. **A.** Graphic outlining key developmental stages of the early human embryo according to the embryo staging carried out by Petropoulos et al. 2016. **B.** Plotting individual embryos (red dots) demonstrates that cell types are largely restricted by developmental stage. **C.** UMAP embedding with cell state annotations. **D.** RNA velocity vectors overlaid onto the UMAP embedding. Samples in grey were not part of the RNA velocity analysis owing to the scRNA-seq spliced and unspliced counts matrices only being available for the Petropoulos et al. 2016 dataset. **E.** Trajectory inference was performed using STREAM (Chen et al. 2019).

Development • Supplementary information

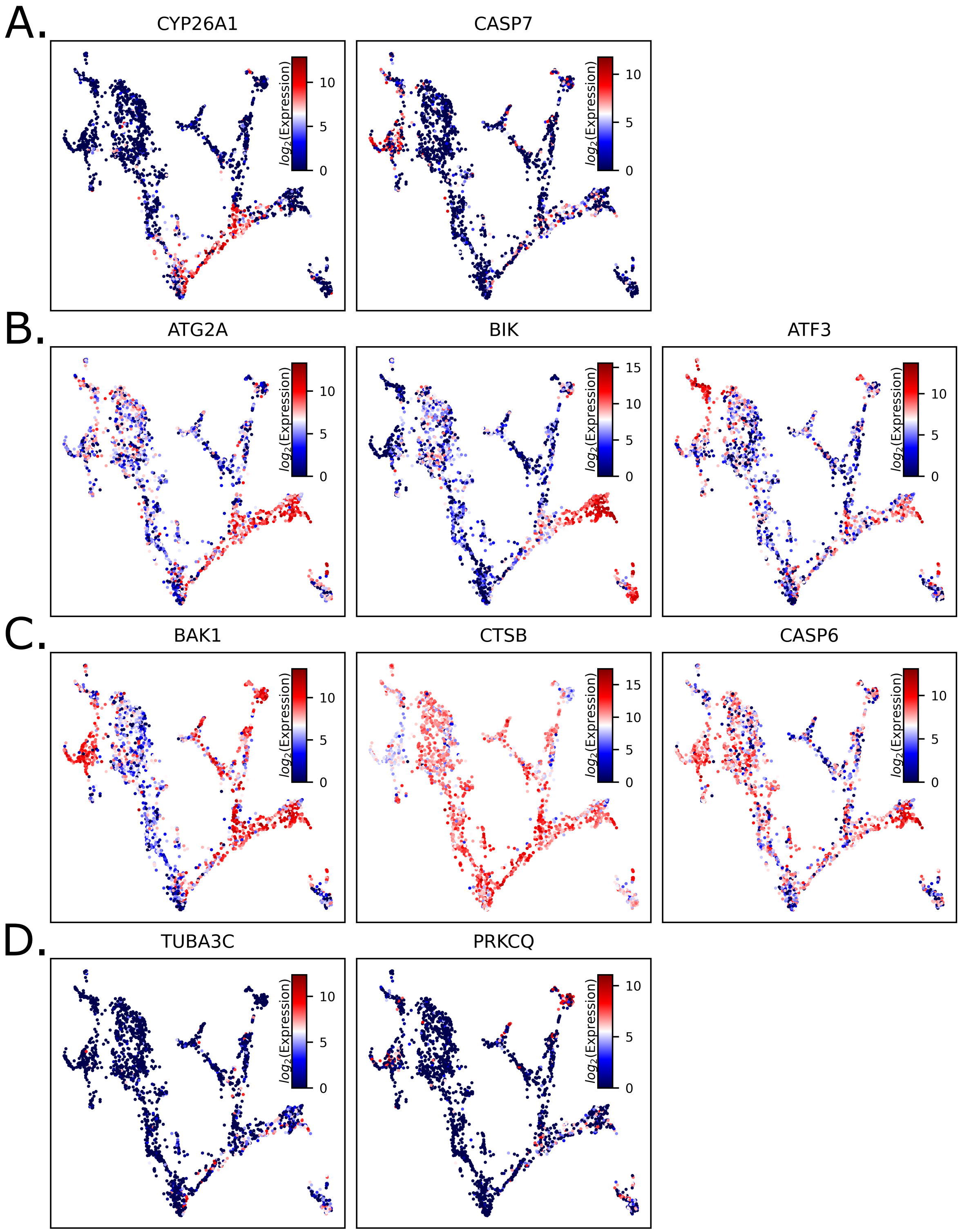

Fig. S7. Proposed apoptotic markers of NCC cells display NCC non-specific expression profiles. Visualisation of NCC specific apoptosis markers proposed by Singh et al. 2023 show a variety of NCC specific and non-specific expression patters. **A.** Proposed apoptosis markers that have relatively specific NCC or ICM/TE branch expression. **C.** Proposed apoptosis markers that show higher expression in morula and/or 8-cell populations than NCC or ICM/TE cells. **C.** Proposed apoptosis markers that are upregulated across several cell types of the day 3-14 human embryo. **D.** Proposed apoptosis markers that show sparse/low expression throughout the day 3-14 human embryo.

Development • Supplementary information

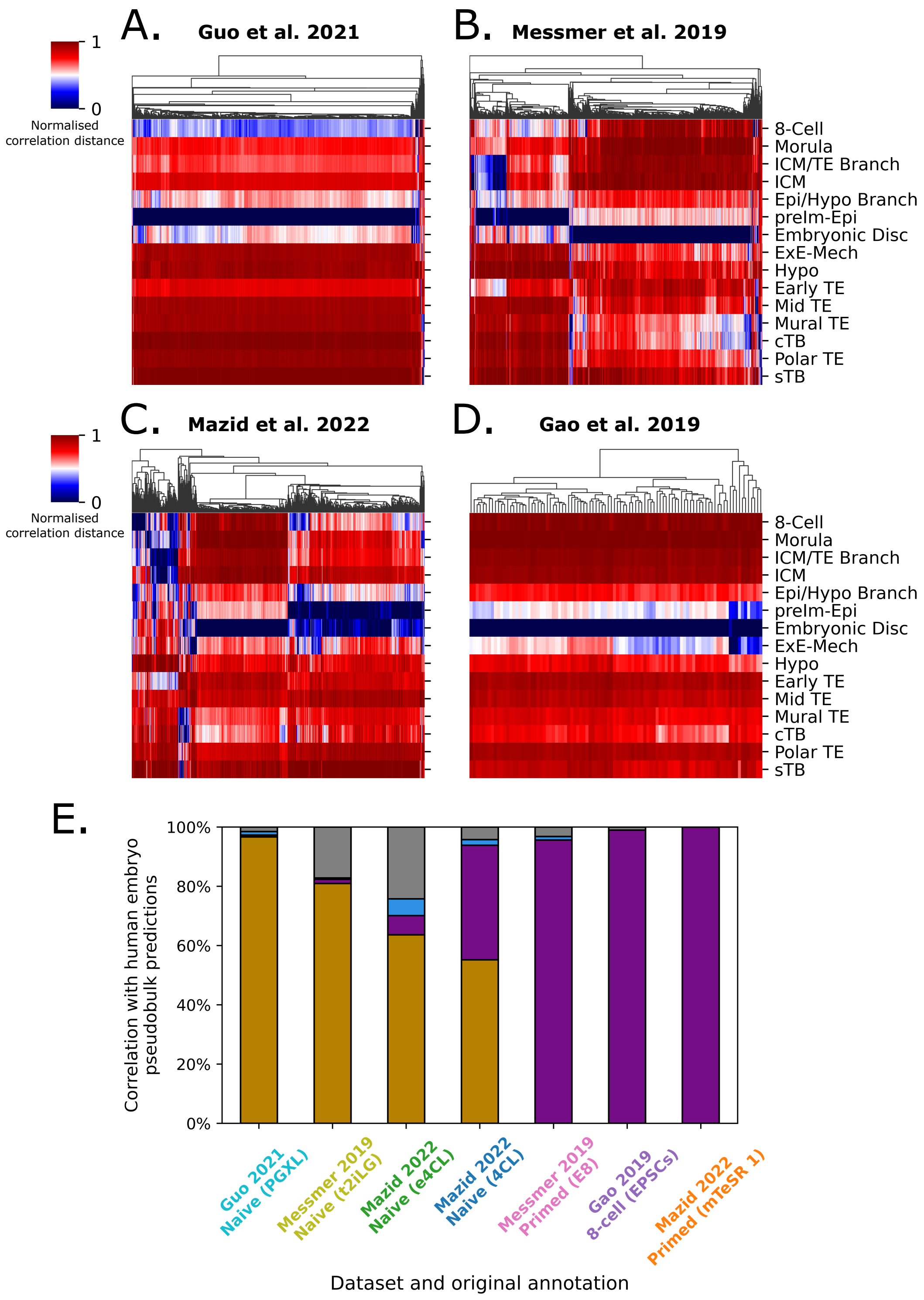

Fig. S8. Human embryo cell type label transfer to pluripotent stem cell culture samples. Related to Fig 5. A-D. Pseudo-bulk for each of our human embryo cell type annotations was generated by taking the average expression of samples within each group, for each gene. The correlation distance metric was then calculated between each of the pseudo-bulk samples and each of the scRNA-seq samples from the (Guo et al. 2021) (A.), (Messmer et al. 2019) (B.), (Mazid et al. 2022) (C.) and (Gao et al. 2019) (D.) PSC cultures conditions. Correlation distances were calculated on the raw expression counts subsetted down to the 3012 genes identified in this manuscript as informative of early human embryo cell identity. For each scRNA-seq sample, the correlation distances were normalised between 0 and 1 to aid visualisation of which human embryo cell type they are most similar to (dark blue). **E.** Bar plots summarising the proportions of which human embryo cell types the PSC scRNA-seq samples are most similar to shows consistency with cell type label transfer performed in the low dimensional UMAP space (Fig 5C).

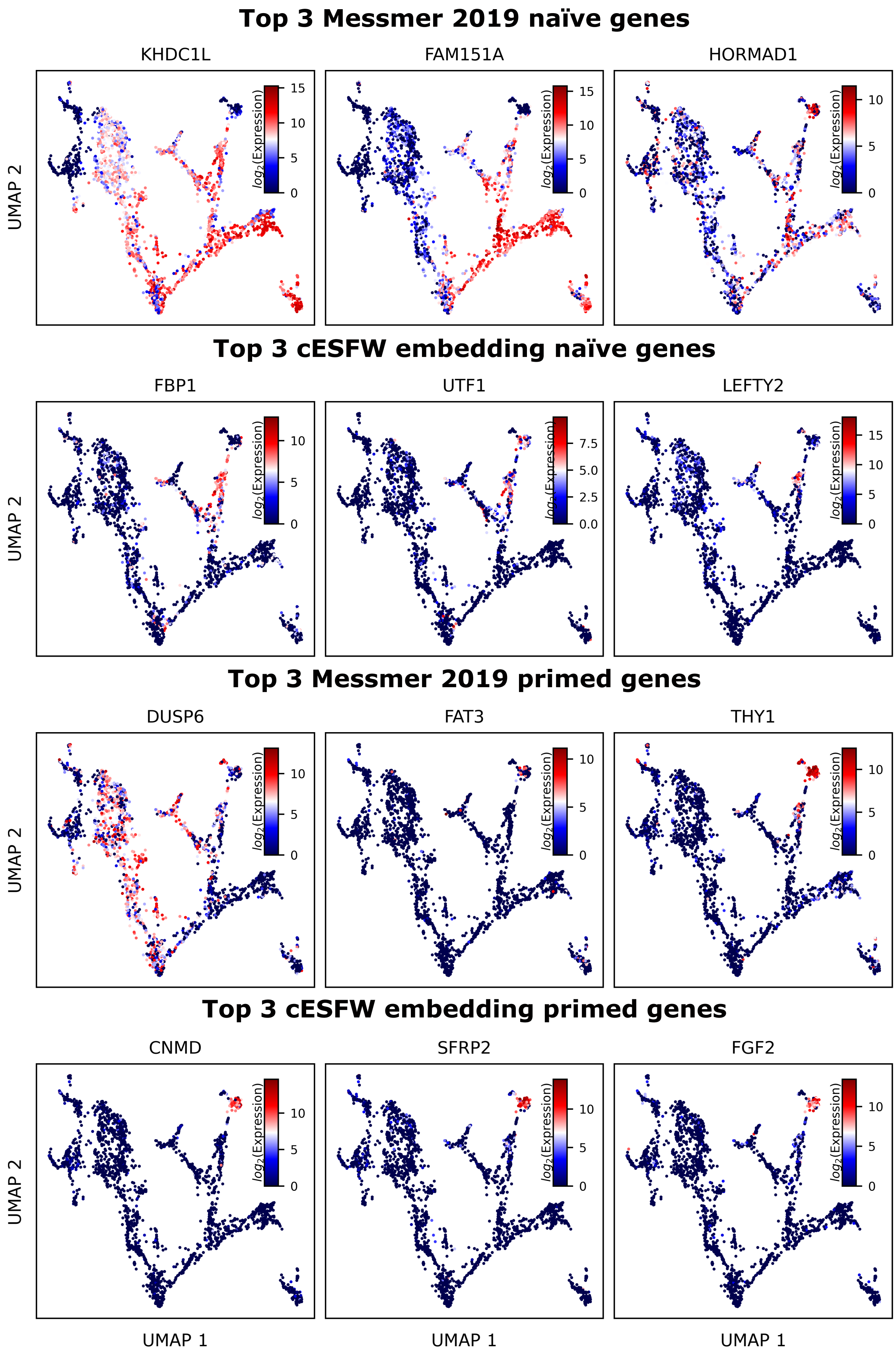

Figure S9. Comparison of top 3 Messmer et al. 2019 naïve and primed genes against the top 3 naïve and primed genes according to our cell type annotation ranked gene lists (Table S1).

Development • Supplementary information

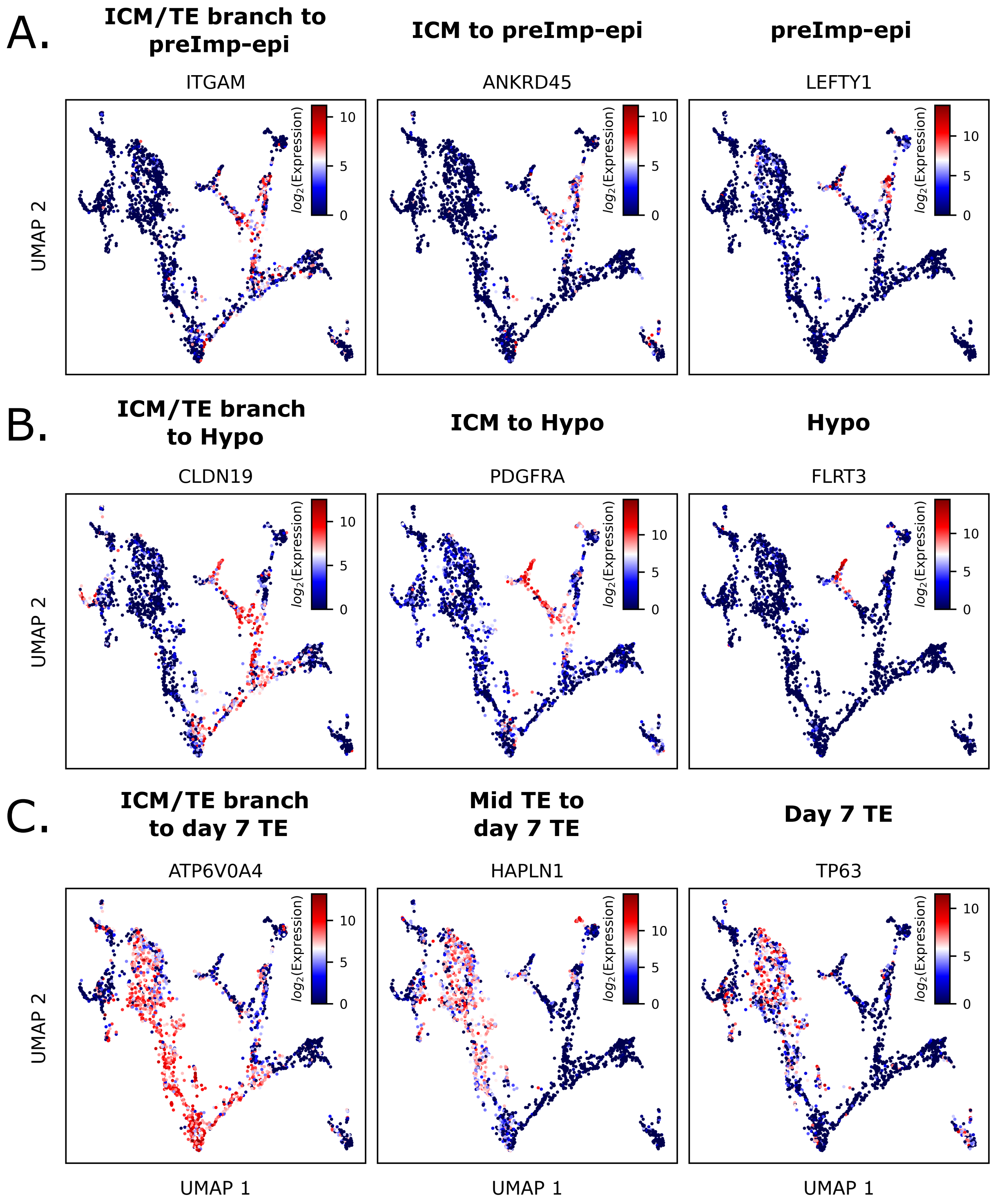

Fig. S10. Emerging epiblast, hypoblast and trophectoderm signatures during blastocyst development. UMAP gene expression profiles for example genes of the Epi (A.), Hypo (B.) or TE (C.) lineages. Presented genes are from those that are highlighted in green in the heatmaps shown in Fig 6.

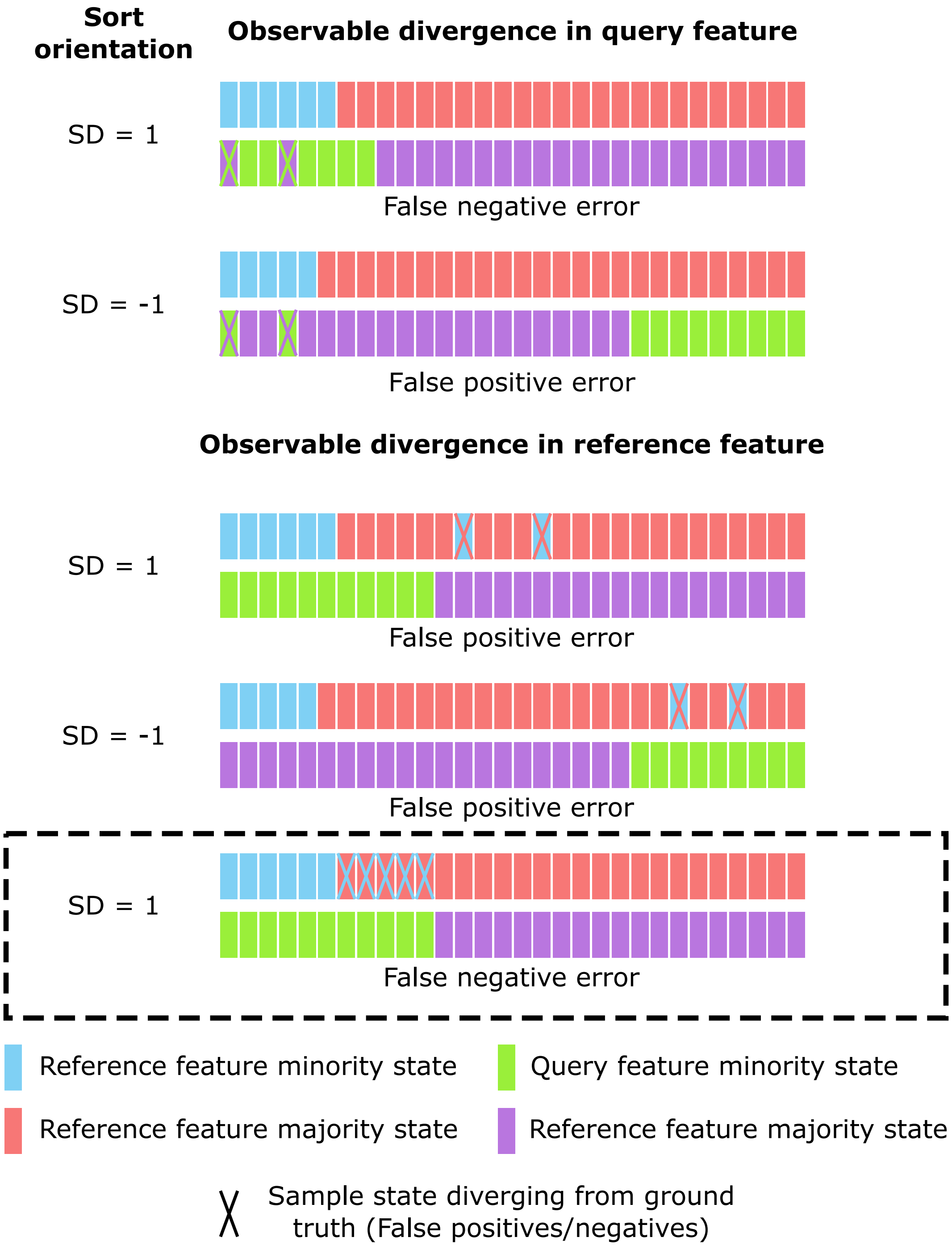

Fig. S11. Entropy sorting error scenarios. A set of simple examples that demonstrate the 5 known error scenarios where expression states between two features can be quantified as potential false positive (FP) or false negative (FN) data points through the ES parabola. SD indicates the split direction for the observed reference feature (RF) and query feature (QF) pair. These examples are set up with discrete states (minority Vs majority) for simplicity, but can be expanded to continuous data, as per SUPPLEMENTARY MATERIALS AND METHODS. The top 4 scenarios were outlined in detail in our previous work (A. Radley et al. 2023). The bottom error scenario (black box) outlines a previously undescribed error scenario where ES divergence indicative of FN expression values can be observed.

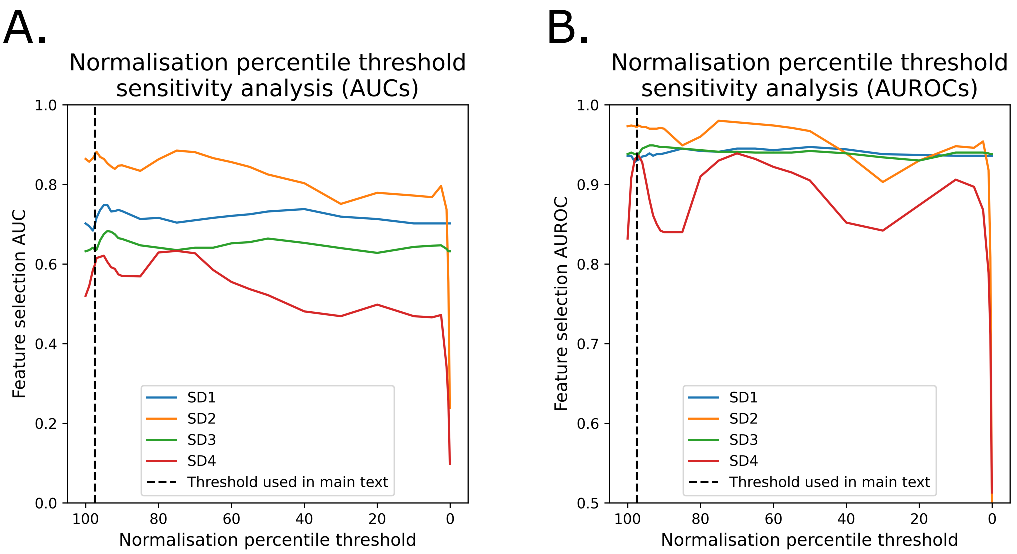

Fig. S12. Feature normalisation percentile sensitivity. A, B. Varying the feature percentile threshold (*p*) for normalisation of features on each of the synthetic datasets shows that the results of cESFW are robust to difference choices of *p*, as demonstrated by minimal changes to the overall AOC (A.) and AUROC (B.) scores generated by cESFW feature ranking.

Development • Supplementary information

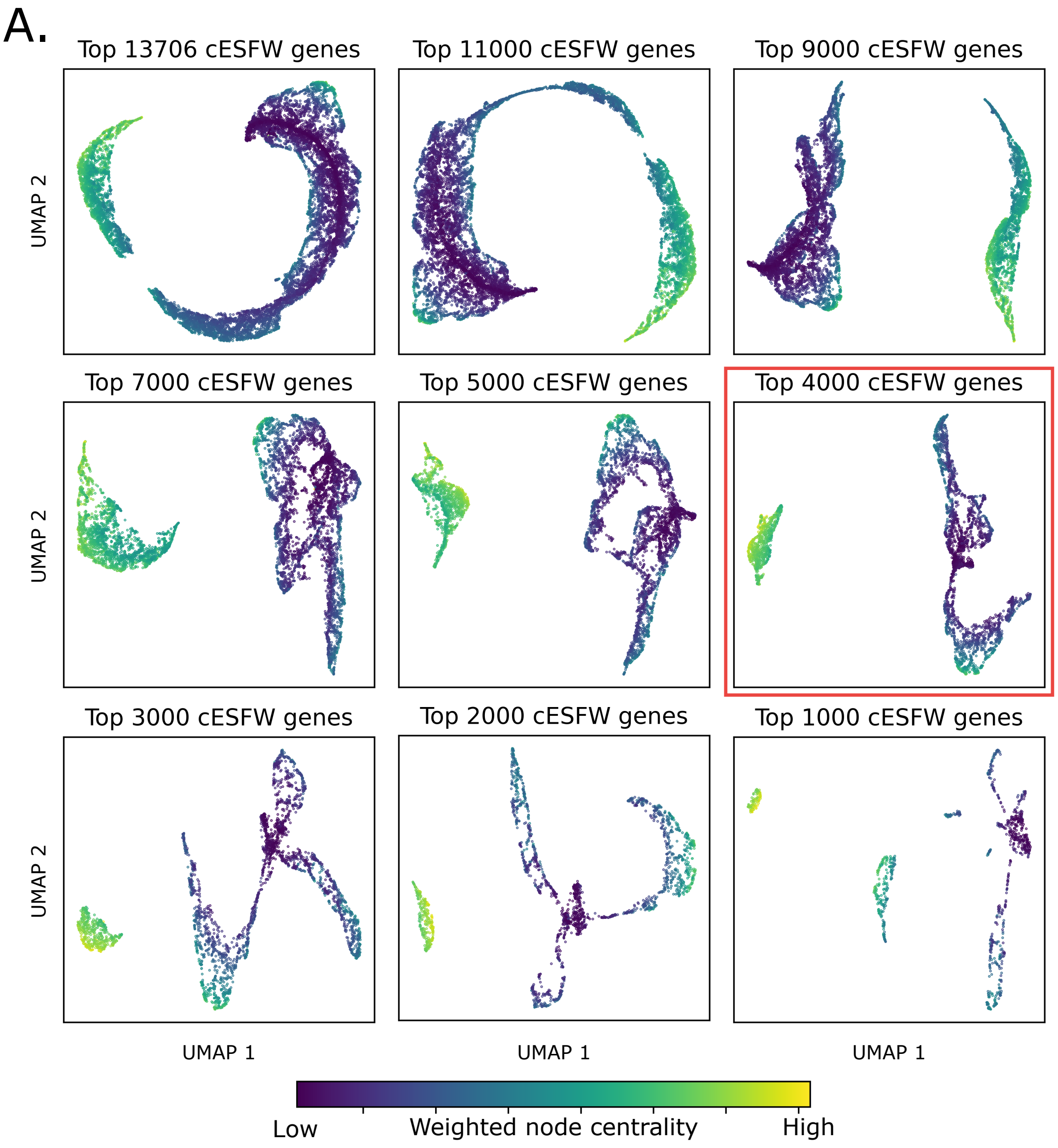

B.

	Gene Ontologies		
1	cell-cell adhesion via plasma-membrane adhesion molecules	16	branching involved in blood vessel morphogenesis
2	extracellular structure organization	17	regulation of endothelial cell proliferation
3	extracellular matrix organization	18	negative regulation of blood coagulation
4	external encapsulating structure organization	19	axon guidance
5	regulation of synapse assembly	20	sodium ion transmembrane transport
6	chemical synaptic transmission	21	heart development
7	anterograde trans-synaptic signaling	22	inorganic cation transmembrane transport
8	heterophilic cell-cell adhesion via plasma membrane cell adhesion molecules	23	endoderm formation
9	skeletal system development	24	neuron differentiation
10	positive regulation of cell junction assembly	25	adenylate cyclase-modulating G protein-coupled receptor signaling pathway
11	synapse organization	26	regulation of ERK1 and ERK2 cascade
12	regulation of gap junction assembly	27	positive regulation of synapse assembly
13	positive regulation of epithelial cell proliferation	28	homophilic cell adhesion via plasma membrane adhesion molecules
14	glial cell differentiation	29	positive regulation of endothelial cell proliferation
15	gonad development	30	defense response to bacterium

C.

	Gene Ontologies		
1	mitochondrial translational elongation	16	aerobic electron transport chain
2	mitochondrial translational termination	17	mitochondrial ATP synthesis coupled electron transport
3	translational elongation	18	cellular macromolecule biosynthetic process
4	translational termination	19	pre-replicative complex assembly
5	mitochondrial translation	20	peptide biosynthetic process
6	gene expression	21	protein-DNA complex assembly
7	ribosome biogenesis	22	regulation of mRNA catabolic process
8	mRNA splicing, via spliceosome	23	RNA processing
9	ncRNA processing	24	mitochondrial electron transport, NADH to ubiquinone
10	rRNA processing	25	RNA splicing
11	mRNA processing	26	antigen processing and presentation of exogenous peptide antigen via MHC class I, TAP-dependent
12	translation	27	regulation of cellular amine metabolic process
13	rRNA metabolic process	28	regulation of mRNA stability
14	RNA splicing, via transesterification reactions with bulged adenosine as nucleophile	29	DNA-dependent DNA replication
15	ribonucleoprotein complex assembly	30	negative regulation of cell cycle G2/M phase transition

Fig. S13. Identifying a cluster of genes with branching structure. A. UMAPs of top ranked genes with increasingly stringent thresholds shows the emergence of a cluster of genes that form a graph with branches. We hypothesise that these branches indicate sets of co-regulating genes. To generate the high resolution human embryo embedding show in the main text of this paper, we used a cESFW selection threshold that took the top 4000 genes according to cESFW weighting (red box in this figure). **B.** Gene ontology analysis of the genes in blue branching cluster of the top 4000 genes (red box) shows that these genes are enriched for developmental and differentiation terms. **C.** Gene ontology analysis of the genes in yellow cluster of the top 4000 genes (red box) shows that these genes are enriched for cell transcription, translation and metabolic processes.

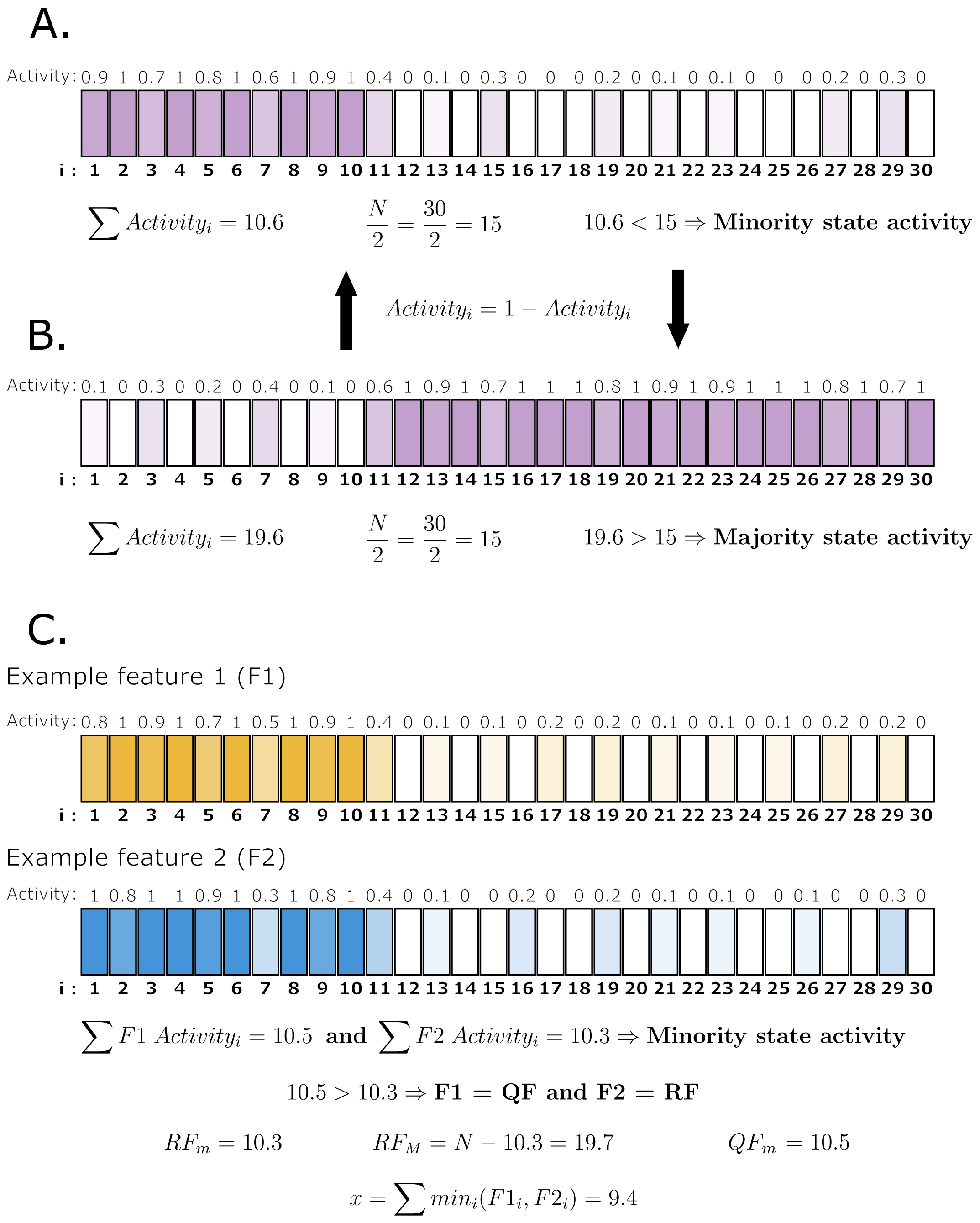

Fig. S14. Calculating the inputs of the ESE for continuous data. A, B. An example feature where the sample activities are represented in gradients of purple. In A. the feature is being observed in a form that represents its minority state activities, since the sum of the activities for all 30 samples is less than $\frac{N}{2} = 15$. We can take the same feature and view it in its majority state activities by deducting the minority state activities of each sample from 1, as shown in B. The majority state form in B. can then be returned to the minority state form in A. by the same process. **C.** A pair of features in the minority state activity forms. Since $\sum F1Activity_i > \sum F2Activity_i$, F1 is the QF. Following this, we can find RFm, RF_M, QF_m and x, so that the CE between F1 and F2 can be calculated via the ESE (Eqn 2).

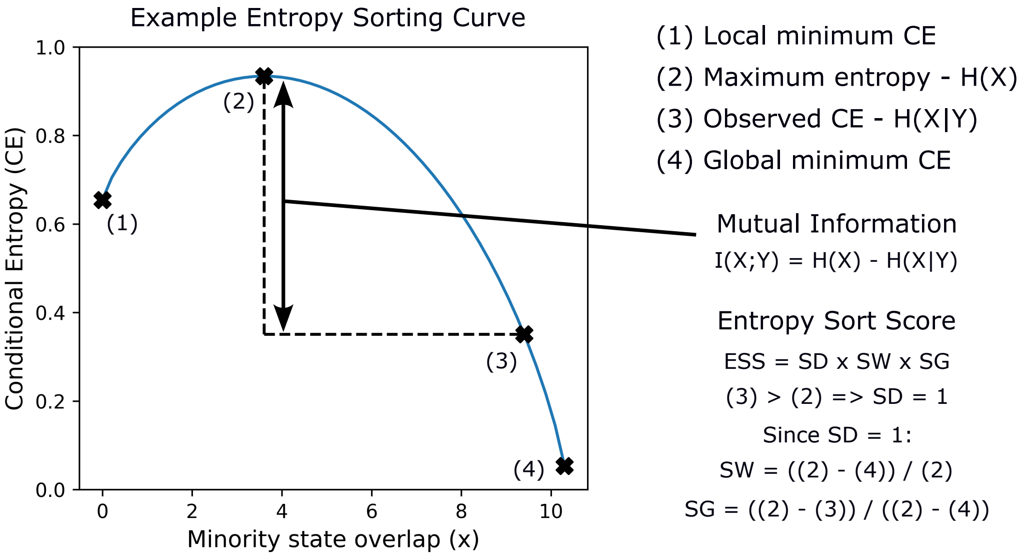

Fig. S15. The relationship between Mutual Information and the ESS. Since (2) = H(X), and (3) = H(X|Y), we can substitute I(X;Y) into the SG equation to demonstrate that the mathematical framework of Entropy Sorting may be thought of as a bounded extension of mutual information. Having an explicitly bounded information theoretic allows us to rationally normalise our correlation metric (ESS) and set up hypothesis tests, while benefiting from properties of information theory, such as both I(X;Y) and the ESS being able to capture non-linear relationships between features/variables.

Table S1. Human embryo scRNA-seq cell type ranked gene lists. Ranked gene lists created using the Entropy Sort Score (ESS) correlation metric to rank the enrichment of each of the 16719 genes in each of the 15 annotated cell types.

Available for download at
https://journals.biologists.com/dev/article-lookup/doi/10.1242/dev.202832#supplementary-data

Table S2. Algorithm 1. Where input *M* is the *i* by *j* raw scRNA-seq counts matrix (*i*=samples/ cells, *j*=genes), *N* is the number of samples/cells comprising (*N*=*j*), and p is the percentile used to clip the maximum expression of each gene. The output, M_m, is the normalised minority state activity *i* by *j* matrix.

```
Algorithm 1: Single cell RNA sequencing minority state normalisation
  Input  : M, p, N
  Output : M_m
  begin
    for Each of j genes in M do
      Find the p^th percentile expression value.
      if p^th percentile > 0 then
        Clip expression values so that values greater than the p^th percentile are equal to the p^th percentile.
        Divide all values by the p^th percentile.
      end
      if ∑Activities_i > N/2 then
        Activities_i = 1 − Activities_i
      end
    end
  end
```

Table S3. Algorithm 2. Where input M_m is the *i* by *j* normalised minority state activity (*i*=samples/ cells, *j*=genes), *N* is the number of samples/cells comprising (*N*=*j*) and *k* is the number of datasets the were combined to create M_m.

```
Algorithm 2: Exclude genes likely contributing to batch effects
  Input  : M_m, N
  Output : A list of features significantly enriched in 1 or more datasets.
  begin
    for Each of k datasets in M_m do
      Create dataset pseudo-feature by creating a vector of 0's length N and then replacing all entries where a sample
       is a member of dataset k to 1.
      if ∑Dataset Activities_i > N/2 then
        Dataset Activities_i = 1 − Dataset Activities_i
      end
      for Each of j genes in M_m do
        Calculate EP of the dataset pseudo-feature with gene j.
      end
    end
    Identify genes with EP > 0 for at least 1 of the k dataset pseudo-features.
  end
```

Development • Supplementary information

Supplementary Materials and Methods

From discrete data Entropy Sorting to continuous data Entropy Sorting

Entropy Sorting (ES) is a mathematical framework that quantifies the correlations between features by reformulating conditional entropy as a sorting problem, rather than a probabilistic one. By imagining the relationships between features as sorting problems, ES gives us access to mathematical properties that are particularly useful when analysing high dimensional data. In A. Radley et al. 2023, the authors comprehensively outline the derivation of ES and demonstrate how properties of ES can be helpful when interrogating high dimensional data. The foundation of ES is the Entropy Sort Equation (ESE), which given two features, calculates their conditional entropy (CE). (A. Radley et al. 2023) defined the ESE as;

$$ESE = \frac{G_1}{N}\left(-\frac{x}{G_1}log_2(\frac{x}{G_1}) - \frac{G_1-x}{G_1}log_2(\frac{G_1-x}{G_1})\right) + \frac{G_2}{N}\left(-\frac{QF_m-x}{G_2}log_2(\frac{QF_m-x}{G_2}) - \frac{G_2-QF_m+x}{G_2}log_2(\frac{G_2-QF_m+x}{G_2})\right), \quad (1)$$

where RF and QF stand for the Reference Feature and Query Feature respectively, and N equals the number of samples that comprise the RF/QF. According to the maximum entropy principle, given two features the QF is designated as the feature with the higher independent entropy, and as such the RF is the feature with the lower independent entropy. A. Radley et al. 2023 derive the ESE from a discrete state scenario where a feature can only be observed in one of two states, i.e. 0 or 1, where 1 is always designated as the minority (m) state. For example if $N = 30$ and we observe 20 samples of a feature to display 1's and 10 to display 0's, we must switch the 0 and 1 values for each sample such that there are instead 10 1's and 20 0's. Following this, G_1 is the number of samples in the RF that are equal to 1 and G_2 equals the number of samples in the RF equal to 0. Hence, $G_1 + G_2 = N$. Likewise, QFm is equal to the number of samples in the QF that display the minority states (i.e. are equal to 1). Finally, x is the only variable in the ESE, and denotes the number of samples where samples both the RF and QF are equal to 1. We call x the overlap between the RF and QF minority states.

An intuitive way of interpreting the ESE is that it quantifies the degree to which the less common observation of two features overlap with one another. In the context of gene expression data, samples displaying 0 could be when a gene is inactive in a cell and those displaying 1 have the gene as active. The ESE then quantifies how well the active states of both genes co-occur with one another. A clear limitation of the above ESE formulation is that many datasets, including gene expression data, consist of more than two states/values. Here we demonstrate how a simple alteration to the ES framework allows us to expand the usage of ES to datasets with continuous values.

We start by noting that rather than calling the 0 and 1 values "states", we can refer to them as activities, where 0 indicates the feature is completely inactive and 1 designates the the feature is fully active. The notion of activity facilitates an interpretable meaning of a sample displaying a value of 0.5 for a feature, which would mean that the feature is 50% active in the given sample, rather than there being a 50/50 chance of observing the state as active/inactive. In the MATERIALS AND METHODS we discuss how we ensure all values in a scRNA-seq dataset are placed with a range of 0-1. For this section, we will use the examples in Figure S14 to redefine each of the terms in the ESE to be conducive with continuous data in the following manner:

- N: Remains as the number of samples comprising the RF/QF.
- $_m$: Minority state activity. In the discrete case, this was the number of samples displaying the minority state. In the continuous case, $_m$ is equal to the sum of all minority state activities. In Figure S14A, $_m = 10.6$.
- $_M$: Majority state activity. In the discrete case, this was the number of samples displaying the majority state. In the continuous case, $_M$ is equal to the sum of all majority state activities. In Figure S14B, $_M = 19.4$.
- QF: Of the two features being compared against one another, the QF is designated as the feature with the larger minority state activity ($_m$). Following this definition, in Figure S14C the QF is F1.
- RF: For a pair of features being compared against one another, the RF is designated as the feature with the smaller minority state activity ($_m$). Following this definition, in Figure S14C the RF is F2. In cases where F1 and F2 have equal cardinalities, one should arbitrarily assign a feature as the RF/QF.
- G_1: In the ESE derived by A. Radley et al. 2023 (Eqn (1)), G_1 is equivalent to RF_m, hence to make nomenclature more consistent, from now on we substitute RF_m in place of G_1. In Figure S14C, $RF_m = 10.3$.
- G_2: In the ESE derived by A. Radley et al. 2023 (Eqn (1)), G_2 is equivalent to RF_M, hence to make nomenclature more consistent, from now on we substitute RF_M in place of G_2. In Figure S14C, $RF_M = 19.7$.
- QF_m: The QF minority state activity. In Figure S14C, QF_m = 10.5.
- x: The minority state activity overlap. In the continuous case, the overlap may be thought of as the sum of the minority

state activities that co-occur in individual samples for both features. Mathematically, this is the minimum value observed in each sample for both features. In Figure S14C, $x = 9.4$.

Substituting these new continuous form definitions into the ESE leaves us with the following equation:

$$ESE = \frac{RF_m}{N}\left(-\frac{x}{RF_m}log_2(\frac{x}{RF_m}) - \frac{RF_m - x}{RF_m}log_2(\frac{RF_m - x}{RF_m})\right) + \frac{RF_M}{N}\left(-\frac{QF_m - x}{RF_M}log_2(\frac{QF_m - x}{RF_M}) - \frac{RF_M - QF_m + x}{RF_M}log_2(\frac{RF_M - QF_m + x}{RF_M})\right), \tag{2}$$

which only varies from Eqn 1 due to the nomenclature substitution of RF_m and RF_M for G_1 and G_2 respectively. Hence we have demonstrated that the above definitions leading to Eqn 2 allow us to apply the principles of ES to continuous data.

Assumed properties of underlying sample distributions

When calculating correlation metrics between features in a dataset, assumptions about the underlying distributions are often applied. For example, Pearson's correlation assumes a linear relationship between features, and the discrete form of the ESE assumed that all samples took values of either 0 or 1. Assumptions such as these limit the generalisability of these metrics. In scRNA-seq data, gene expression values are not limited to 0 and 1, and the relationships between genes can be non-linear.

The continuous form of the ESE (Eqn 2) overcomes both of these challenges. We have already outlined how Eqn 2 incorporates values other than 0 and 1. With regards to non-linear/skewed relationships, we note that the ESS is closely related to the commonly used correlation metric, Mutual Information (MI). An advantage of MI over other correlation metrics is that it is well suited for capturing non-linear relationships between features. With regards to the ESE, the relationship between MI and the ESS can be visualised on an ES parabola to show that the ESS is a bounded/normalised interpretation of MI (Fig S15). In other words, ES can be thought of as providing a mathematically principled way to scale relationships between features via the explicitly defined boundaries of the system. Crucially, since the ESS is an extension of MI, ES and the ESS are also well suited for capturing non-linear relationships between features.

The main assumption for applying ES is that the features can be meaningfully scaled between values of 0 and 1. For gene expression, an intuitive way of achieving this is to inspect each gene and designate 0 count values as having 0 expression activity, and the maximum counts as having activities of 1, and all values in between existing within the [0,1] interval. A useful property of ES is that we do not need to assume a particular shape or distribution of the samples within the [0, 1] interval. The ES framework is non-parametric and does not require an assumed distribution to calculate the conditional entropy (CE), even in the continuous form. This is possible because the ES framework is formulated by turning the probabilistic form of CE into an ordinary differential equation (ODE), where the only dependent variable, x, is the overlap between the minority state activities of each individual sample. This calculation is explicitly identifiable/calculable, and is permutation invariant, meaning the shape of the distributions of a reference feature (RF) and query feature (QF) does not need to be assumed/defined. In other words, the ES framework quantifies to what degree active expression states enrich/overlap with one another in a manner that is robust to different distribution shapes. We describe the normalisation procedure used in this manuscript in the MATERIALS AND METHODS, but ultimately this procedure is dataset dependent and left to the user to determine the best approach.

References

Chen, H. et al. (2019). “Single-cell trajectories reconstruction, exploration and mapping of omics data with STREAM”. *Nature Communications 2019 10:1* 10.1, pp. 1–14.

Corujo-Simon, E., A. H. Radley, and J. Nichols (2023). “Evidence implicating sequential commitment of the founder lineages in the human blastocyst by order of hypoblast gene activation”. *Development (Cambridge)* 150.10.

Gao, X. et al. (2019). “Establishment of porcine and human expanded potential stem cells”. *Nature Cell Biology 2019 21:6* 21.6, pp. 687–699.

Guo, G. et al. (2021). “Human naive epiblast cells possess unrestricted lineage potential”. *Cell Stem Cell* 28.6, pp. 1040–1056.

Li, Z., O. Kurosawa, and H. Iwata (2019). “Establishment of human trophoblast stem cells from human induced pluripotent stem cell-derived cystic cells under micromesh culture”. *Stem Cell Research & Therapy* 10.1.

Liu, D. et al. (2022). “Primary specification of blastocyst trophectoderm by scRNA-seq: New insights into embryo implantation”. *Science Advances* 8.31, p. 3725.

Mazid, M. A. et al. (2022). “Rolling back human pluripotent stem cells to an eight-cell embryo-like stage”. *Nature 2022 605:7909* 605.7909, pp. 315–324.

Meistermann, D. et al. (2021). “Integrated pseudotime analysis of human pre-implantation embryo single-cell transcriptomes reveals the dynamics of lineage specification”. *Cell Stem Cell* 28.9, pp. 1625–1640.

Messmer, T., F. von Meyenn, A. Savino, F. Santos, H. Mohammed, A. T. L. Lun, J. C. Marioni, and W. Reik (2019). “Transcriptional Heterogeneity in Naive and Primed Human Pluripotent Stem Cells at Single-Cell Resolution”. *Cell Reports* 26.4, p. 815.

Petropoulos, S., D. Edsgärd, B. Reinius, Q. Deng, S. P. Panula, S. Codeluppi, A. Plaza Reyes, S. Linnarsson, R. Sandberg, and F. Lanner (2016). “Single-Cell RNA-Seq Reveals Lineage and X Chromosome Dynamics in Human Preimplantation Embryos”. *Cell* 165.4, pp. 1012–1026.

Radley, A., E. Corujo-Simon, J. Nichols, A. Smith, and S. J. Dunn (2023). “Entropy sorting of single-cell RNA sequencing data reveals the inner cell mass in the human pre-implantation embryo”. *Stem Cell Reports* 18.1, pp. 47–63.

Singh, M. et al. (2023). “A new human embryonic cell type associated with activity of young transposable elements allows definition of the inner cell mass”. *PLOS Biology* 21.6, e3002162.

Stirparo, G. G., T. Boroviak, G. Guo, J. Nichols, A. Smith, and P. Bertone (2018). “Integrated analysis of single-cell embryo data yields a unified transcriptome signature for the human pre-implantation epiblast”. *Development (Cambridge)* 145.3.

Taubenschmid-Stowers, J., M. Rostovskaya, F. Santos, S. Ljung, R. Argelaguet, F. Krueger, J. Nichols, and W. Reik (2022). “8C-like cells capture the human zygotic genome activation program in vitro”. *Cell Stem Cell* 29.3, p. 449.

Yabe, S., A. P. Alexenko, M. Amita, Y. Yang, D. J. Schust, Y. Sadovsky, T. Ezashi, and R. M. Roberts (2016). “Comparison of syncytiotrophoblast generated from human embryonic stem cells and from term placentas”. *Proceedings of the National Academy of Sciences of the United States of America* 113.19, E2598–E2607.

Yang, R. et al. (2021). “Amnion signals are essential for mesoderm formation in primates”. *Nature Communications 2021 12:1* 12.1, pp. 1–14.

Yue, C., A. C. H. Chen, S. Tian, S. W. Fong, K. C. Lee, J. Zhang, E. H. Y. Ng, K. F. Lee, W. S. B. Yeung, and Y. L. Lee (2020). “Human embryonic stem cell–derived blastocyst-like spheroids resemble human trophectoderm during early implantation process”. *Fertility and Sterility* 114.3, pp. 653–664.

Zadora, J. et al. (2017). “Disturbed placental imprinting in preeclampsia leads to altered expression of DLX5, a human-specific early trophoblast marker”. *Circulation* 136.19, pp. 1824–1839.
