# Supplementary material for: Branching topology of the human embryo transcriptome revealed by Entropy Sort Feature Weighting
Source: Development. 2024 Jun 13;151(11):dev202832. doi: 10.1242/dev.202832 (PMC11213519; doi:10.1242/dev.202832)
Supplement: Supplementary file 1 [file develop-151-202832_review_history.pdf]

Branching topology of the human embryo transcriptome revealed by Entropy Sort Feature Weighting

Arthur Radley, Stefan Boeing and Austin G Smith
DOI: 10.1242/dev.202832

Editor: Anna-Katerina Hadjantonakis

Review timeline

Submission to Review Commons:	24 October 2023
Original submission:	26 February 2024
Editorial decision:	12 April 2023
First revision received:	23 April 2023
Accepted:	24 April 2023

Reviewer 1

Evidence, reproducibility and clarity

Summary

In this manuscript, Arthur Radley and Austin Smith designed a new feature selection method for scRNA-Seq, which is a successor to ESFW previously proposed by the same authors. As an evolution of this earlier framework, cESFW is also based on the idea that informative genes share information with other genes, whereas non-informative genes have a more random relative expression. The authors emphasize the key importance of feature selection in the scRNA-Seq workflow and assess the current state of the art for this step. They also propose that better feature selection leads to less data transformation. They show that cESFW outperforms Scran and Seurat feature selection in most cases of synthetic datasets. cESFW is then used in the context of early human development, re-analysing data from several published datasets where they show that they do not require batch correction. They also further strengthen the conclusion that a "2-step" model for TE-ICM and EPI-Hyp differentiation is also present in human embryos. Finally, they map several types of in vitro pluripotent stem cells, in particular primed and naive, to their manifold and study the evolution of the gene signatures during early human development.

Overall, the manuscript is well written and presents a solid methodology. The re-analysis of human early development is convincing and justified. The main critic is that the quality of figures can be greatly improved: their resolution is too low and they are hard to read. For instance, more contrasted color schemes could be used to improve clarity, and given the high number of clusters for some UMAPs, indicating the name of some cluster near their centroids should improve clarity.

Comments:
Page 2
I think the criticism of PCA is unfair because it is not a true feature selection method, and it is mainly used for computational purposes. I believe that for most workflows, between 30 and 50 PCs are retained, which do not significantly change the results in the downstream analyses. The citation (Yeung and Ruzzo 2001) does not seem appropriate, as they examine cases where only a small number of PCs are retained, outside the context of scRNA-seq.

"Furthermore, HVG selection has been found to be biased toward selecting highly expressed genes over low expressed genes." Could the author justify or remove this statement, as the Seurat and

© 2024. Published by The Company of Biologists under the terms of the Creative Commons Attribution License (https://creativecommons.org/licenses/by/4.0/).

Scran methods are specifically designed to consider average expression to determine HVG? The cited article (Yip, Sham, and Wang 2019) raises this issue for methods other than Seurat and scran.

Page 6
I might have missed it, but I do not understand the number of cells in the early human development dataset also shown in Figure S2B. The Petropoulos et al. dataset alone is larger than the sum of cells from different cell types. Is there some filtering step that is not described?
The unsupervised clustering used to annotate cell types is unconventional (especially with the high number of clusters chosen), which is not a problem, but should be clarified. Improving the figure 3D to make it clearer and providing a cell cluster correlation plot might help to better appreciate the relationship between cell types.
It could be emphasized that the ICM/TE branch cell type is a major difference with the mouse topology, as the readers might not be aware that the ICM/TE is an unspecified blastocyst state that only exists in humans.

Page 9
To further substantiate the stepwise ICM/TE and EPI/PrE specification events, authors could project cells from each embryo on the UMAP, and analyze what are the co-occurrence of cells (as performed for instance in Meistermann et al 2021). This should show as reported (and cited by the authors) that some GATA3 positive cells (TE fated) start appearing from late morula stage and that ICM cells almost never co-exist with EPI nor Hyp in embryos.

Significance

The presented methodology shows significant value especially in the field of scRNA-Seq, where the critical step of feature selection is often inadequately addressed. Furthermore, this field is characterized by a limited set of feature selection methodologies. cESFW appears to be an important alternative to HVG methods that could improve scRNA-Seq analysis in certain contexts.

The new findings on early human development are somehow incremental, but a welcome addition to solidify the two-step model and refine the concept of reject cells. The audience for this early development context is specialized, but cESFW will most likely have an impact to the entire field of scRNA-Seq analysis.

Reviewer 2

Evidence, reproducibility and clarity

Here, Radley and Austin present a novel approach for feature weighting in scRNAseq data based on entropy sorting. Feature selection is a central part of scRNAseq analysis, and it is most likely the case that there is no single approach that outperforms all others across all datasets. Hence, innovation in this space is needed for the field. The cESFW method presented here has several appealing properties from a theoretical point of view, and it also performs well on the synthetic and real datasets considered. Nevertheless, there are several major issues that need to be addressed before I can recommend the manuscript for publication:

1. The original entropy sorting (eq 1 in SI 1) is based on only two discrete states. However, calculating entropy for continuous distributions can be more tricky and it is unclear to me what assumptions are made regarding the gene expression. Could the authors clarify what properties of the distribution are required for the updated ESE equation to be valid? Is the only assumption that values are drawn from the [0, 1] interval? What happens if values are highly skewed, ie forming a bimodal or power-law distribution rather than something close to a uniform distribution?
2. How robust is the procedure for the choice of percentile for normalizing the gene expression scores? Does one get roughly the same results for 90-99th percentile or is it sensitive to this choice?
3. Similarly, I am concerned about the procedure for how to choose the number of significant genes. How robust is this process? Also, it is not altogether clear how to generalize the procedure outlined on p19. Most potential users would benefit from more quantitative guidelines. In particular, having to rely on interpretation of GO terms typically requires a considerable amount of understanding about the system at hand which could make it challenging to apply the procedure for

© 2024. Published by The Company of Biologists under the terms of the Creative Commons Attribution License (https://creativecommons.org/licenses/by/4.0/).

others. For most users it would be helpful to know how robust the procedure is to this step and also if there could be more stringent guidelines for how to decide which genes to include.
4. The comparison of the clusterings on p6 is not really fair is it? If I understand it correctly, the 3,012 genes identified by cESFW was used to define clusters in fig 3c through unsupervised clustering. The authors then use HVG methods to identify 3,012 genes and then carries out clustering based on those. To evaluate the methods the silhouette score is used, but the labels from the cESFW clustering is used as ground truth. This does not sound like a fair way to compare. Could the authors please clarify, and if needed come up with an approach where the three methods have a more level playing field if needed.
5. The main cESFW.py file in the github repository is clearly well structured and commented. However, I would like to see a much better documentation so that one does not have to go through the source code to understand what functions there are and what they do. In particular, I would like to see a vignette to make it easier for others to incorporate cESFW into their workflows.

Minor:

Why are the figures not always in order? For example, fig S10 is mentioned before fig S2 on p 6

I am not sure if the indexing in eq 1 (p 18) is correct. j is both on the LHS and it is also being summed over on the RHS. Should one of these be i instead?

Significance

The work presents a new method for feature selection in scRNAseq. Feature selection is a very important step and can have a big impact on findings. The method presented here is theoretically sound and it seems to provide interesting result when applied to early embryo development. However, as cESFW is only tested for one dataset it is unclear how well the method generalizes to other problems and datasets.

My expertise is in computational genomics.

<u>Author response to reviewers' comments</u>

1. General Statements [optional]

This section is optional. Insert here any general statements you wish to make about the goal of the study or about the reviews.

We thank the reviewers for constructive comments and helpful suggestions which we have adopted to clarify and improve the manuscript. In addition, we have added a link to a web portal that will allow readers to visualise gene expression profiles and create their own plots using our early human embryo UMAP embedding (https://bioinformatics.crick.ac.uk/shiny/users/boeings/radley2024umap_app/). Stefan Boeing created this tool and is added to the author list with agreement of other authors.

2. Point-by-point description of the revisions

Reviewer #1 (Evidence, reproducibility and clarity (Required)):

Summary

In this manuscript, Arthur Radley and Austin Smith designed a new feature selection method for scRNA-Seq, which is a successor to ESFW previously proposed by the same authors. As an evolution of this earlier framework, cESFW is also based on the idea that informative genes share information with other genes, whereas non-informative genes have a more random relative expression. The authors emphasize the key importance of feature selection in the scRNA-Seq workflow and assess

© 2024. Published by The Company of Biologists under the terms of the Creative Commons Attribution License (https://creativecommons.org/licenses/by/4.0/).

the current state of the art for this step. They also propose that better feature selection leads to less data transformation. They show that cESFW outperforms Scran and Seurat feature selection in most cases of synthetic datasets. cESFW is then used in the context of early human development, re-analysing data from several published datasets where they show that they do not require batch correction. They also further strengthen the conclusion that a "2-step" model for TE-ICM and EPI-Hyp differentiation is also present in human embyros. Finally, they map several types of in vitro pluripotent stem cells, in particular primed and naive, to their manifold and study the evolution of the gene signatures during early human development.

Overall, the manuscript is well written and presents a solid methodology. The re-analysis of human early development is convincing and justified. The main critic is that the quality of figures can be greatly improved: their resolution is too low and they are hard to read. For instance, more contrasted color schemes could be used to improve clarity, and given the high number of clusters for some UMAPs, indicating the name of some cluster near their centroids should improve clarity.

We agree that the resolution of the figures should be improved. We had to compress the images to satisfy the size limit for uploaded documents to bioRxiv. Our final submission will be of higher quality (original figures are at 900dpi). With regards to colour schemes, this is a surprisingly difficult problem. We tried multiple colour palettes but could not achieve greater contrast. The suggestion to add key cluster names near to their centroids on the UMAPs is an excellent idea, which we have implemented.

Comments:

Page 2
I think the criticism of PCA is unfair because it is not a true feature selection method, and it is mainly used for computational purposes. I believe that for most workflows, between 30 and 50 PCs are retained, which do not significantly change the results in the downstream analyses. The citation (Yeung and Ruzzo 2001) does not seem appropriate, as they examine cases where only a small number of PCs are retained, outside the context of scRNA-seq.

We agree that the criticism of PCA is insufficiently justified by the citation. We thank the reviewer for pointing this out and have removed the comment.

"Furthermore, HVG selection has been found to be biased toward selecting highly expressed genes over low expressed genes." Could the author justify or remove this statement, as the Seurat and Scran methods are specifically designed to consider average expression to determine HVG? The cited article (Yip, Sham, and Wang 2019) raises this issue for methods other than Seurat and scran.

The reviewer is correct that the provided citation highlights Seurat and Scran HVG selection as relatively insensitive to the average gene expression levels compared with other HVG selection methods. We again thank the reviewer and have deleted the comment.

More generally, we have shortened the introduction, focusing on cESFW as a new approach to feature selection rather than critiquing alternative methods.

Page 6
I might have missed it, but I do not understand the number of cells in the early human development dataset also shown in Figure S2B. The Petropoulos et al. dataset alone is larger than the sum of cells from different cell types. Is there some filtering step that is not described?

We have added text in the data availability section to clarify the cells used in our analysis: “The pre-implantation raw counts scRNA-seq data from Yan et al. 2013, Petropoulos et al. 2016, Fogarty et al. 2017, and Meistermann et al. 2021, were compiled into a single gene expression matrix by Meistermann et al. 2021. For information regarding quality control and cell filtering of these 4 datasets, please refer to Meistermann et al. 2021.”

The unsupervised clustering used to annotate cell types is unconventional (especially with the high number of clusters chosen), which is not a problem, but should be clarified. Improving the figure 3D

© 2024. Published by The Company of Biologists under the terms of the Creative Commons Attribution License (https://creativecommons.org/licenses/by/4.0/).

to make it clearer and providing a cell cluster correlation plot might help to better appreciate the relationship between cell types.

We agree that the gene expression heatmap in figure 3D contributed little to the interpretation of the data/results. As suggested, we have replaced this heatmap with a cell cluster correlation plot to help appreciate cell state similarities. (Changes in figure 3.)

It could be emphasized that the ICM/TE branch cell type is a major difference with the mouse topology, as the readers might not be aware that the ICM/TE is an unspecified blastocyst state that only exists in humans.

There appears to be some misunderstanding around the use of “ICM/TE branch”. The cluster comprises an uncommitted population at the branching point from morula to either ICM or TE, as also described in the mouse embryo. We have adjusted the discussion to make more clear that the two branching point clusters are heterogeneous populations, not unitary cell types or states: “The branching populations reside at critical junctures in blastocyst formation, the partitioning of extraembryonic and embryonic lineages. These branchpoint clusters do not define unitary states. On the contrary, cells in these clusters are heterogeneous and may become specified to alternative fates. For example, PDGFRA, a hypoblast marker (Corujo-Simon et al. 2023), and NANOG, an epiblast marker (Allegre et al. 2022), are heterogeneously distributed in the Epi/Hyp branching population. Furthermore, branch cluster boundaries extend beyond the topological bifurcation, potentially indicating that cells remain plastic and may be redirected. This would be consistent with the demonstration in mouse embryos that cells expressing ICM genes remain capable of generating TE up to the late 32-cell stage (Posfai et al. 2017).”

Page 9
To further substantiate the stepwise ICM/TE and EPI/PrE specification events, authors could project cells from each embryo on the UMAP, and analyze what are the co-occurrence of cells (as performed for instance in Meistermann et al 2021). This should show as reported (and cited by the authors) that some GATA3 positive cells (TE fated) start appearing from late morula stage and that ICM cells almost never co-exist with EPI nor Hyp in embryos.

We appreciate this suggestion. We have generated the requested plots showing where cells from individual embryos at different developmental timepoints are positioned on our UMAP embedding. (new supplemental figure (New figure, Figure S6). We present a summary heatmap of cell co-occurrence in revised Figure 4. These results offer greater insight than the RNA velocity analysis, which we have moved to supplemental Figure S6. We have added discussion of these analyses in the “Lineage branching blastocyst development” Results section.

Reviewer #1 (Significance (Required)):

The presented methodology shows significant value especially in the field of scRNA-Seq, where the critical step of feature selection is often inadequately addressed. Furthermore, this field is characterized by a limited set of feature selection methodologies. cESFW appears to be an important alternative to HVG methods that could improve scRNA-Seq analysis in certain contexts.

The new findings on early human development are somehow incremental, but a welcome addition to solidify the two-step model and refine the concept of reject cells. The audience for this early development context is specialized, but cESFW will most likely have an impact to the entire field of scRNA-Seq analysis.

Reviewer #2 (Evidence, reproducibility and clarity (Required)):

Here, Radley and Austin present a novel approach for feature weighting in scRNAseq data based on entropy sorting. Feature selection is a central part of scRNAseq analysis, and it is most likely the case that there is no single approach that outperforms all others across all datasets. Hence, innovation in this space is needed for the field. The cESFW method presented here has several appealing properties from a theoretical point of view, and it also performs well on the synthetic

© 2024. Published by The Company of Biologists under the terms of the Creative Commons Attribution License (https://creativecommons.org/licenses/by/4.0/).

and real datasets considered. Nevertheless, there are several major issues that need to be addressed before I can recommend the manuscript for publication:

1 The original entropy sorting (eq 1 in SI 1) is based on only two discrete states. However, calculating entropy for continuous distributions can be more tricky and it is unclear to me what assumptions are made regarding the gene expression. Could the authors clarify what properties of the distribution are required for the updated ESE equation to be valid? Is the only assumption that values are drawn from the [0, 1] interval? What happens if values are highly skewed, ie forming a bimodal or power-law distribution rather than something close to a uniform distribution?

We agree that it is beneficial to clarify these points. We have added a section titled “Assumed properties of underlying sample distributions” to the supplemental information. Briefly, we show that the ESS correlation metric is directly linked to the commonly used correlation metric, Mutual Information (MI). A desirable properly of MI is that it is able to capture non-linear/skewed relationships between features. The ES framework and ESS share this property with MI, allowing the ES framework to be relatively robust to presence of non-uniform distributions.

The main assumption for applying ES is that the features can be meaningfully scaled between values of 0 and 1. For gene expression, an intuitive way of achieving this is to inspect each gene and designate 0 count values as having 0 expression activity, and the maximum counts as having activities of 1, and all values in between existing within the [0,1] interval. A useful property of ES is that we do not need to assume a particular shape or distribution of the samples within the [0, 1] interval. The ES framework is non-parametric and does not require an assumed distribution to calculate the conditional entropy (CE), even in the continuous form. This is possible because the ES framework is formulated by turning the probabilistic form of CE into an ordinary differential equation (ODE), where the only dependent variable, x, is the overlap between the minority state activities of each individual sample. This calculation is explicitly identifiable/calculable, and is permutation invariant, meaning the shape of the distributions of a reference feature (RF) and query feature (QF) does not need to be assumed/defined. In other words, the ES framework quantifies to what degree active expression states enrich/overlap with one another in a manner that is robust to different distribution shapes.

2 How robust is the procedure for the choice of percentile for normalizing the gene expression scores? Does one get roughly the same results for 90-99th percentile or is it sensitive to this choice?

We have carried out a sensitivity analysis on the choice of percentile for each of the synthetic datasets and added it to the manuscript. (New figure, Figure S11). We find that on each of our 4 synthetic datasets the final results of cESFW are robust to a wide range of normalisation percentiles.

3 Similarly, I am concerned about the procedure for how to choose the number of significant genes. How robust is this process? Also, it is not altogether clear how to generalize the procedure outlined on p19. Most potential users would benefit from more quantitative guidelines. In particular, having to rely on interpretation of GO terms typically requires a considerable amount of understanding about the system at hand which could make it challenging to apply the procedure for others. For most users it would be helpful to know how robust the procedure is to this step and also if there could be more stringent guidelines for how to decide which genes to include.

We understand the reviewers concern regarding the robustness of feature selection on real scRNA-seq datasets. We have now applied our cESFW workflow to peripheral blood mononuclear cells (PBMC) scRNA-seq data, and found cESFW feature selection to be comparable, and by one metric more robust, than Seurat and Scran HVG selection (New Figure S2).

As cESFW is applied to more scRNA-seq data, we will learn more about how results compare to highly variable gene selection, and how workflows may be adapted to optimise results in different scenarios. For example, we have found that supervising the selection of gene clusters using a small set of markers known to be important in the system of study can help identify which clusters of

© 2024. Published by The Company of Biologists under the terms of the Creative Commons Attribution License (https://creativecommons.org/licenses/by/4.0/).

genes should be retained during gene selection. We have added this to the materials and methods with the following paragraph:
"Furthermore, we suggest supervising the selection of gene clusters using a small set of markers known to be important in the system of study. In this work, we found that genes known to be important during early human embryo development (FigS4) are enriched in the dark blue cluster of genes, further suggesting that this cluster of genes is more likely to separate cell type identities in downstream analysis."

While gene cluster selection supervision in this manner requires a degree of domain expertise, we believe this is not unreasonable for most applications, and is the case for many scRNA-seq analysis pipelines.

Our primary software contribution is the cESFW algorithm which calculates the ESS and EP matrices. With this manuscript we provide 6 commented workflows for applying cESFW to different datasets (4 synthetic data, human embryo data, PBMC data). We believe these workflows provide a good balance of documented use cases and user flexibility for cESFW usage. This is important because it is advantageous to be able easily to adapt workflows to incorporate domain expertise and different methodologies. Although workflows such as Seurat and Scran are user-friendly, their rigidity can be difficult when wanting to deviate from their standard workflows. In summary, we believe that our provided workflows are suitable for users to implement cESFW, while providing the flexibility to apply adapted pipelines.

4 The comparison of the clusterings on p6 is not really fair is it? If I understand it correctly, the 3,012 genes identified by cESFW was used to define clusters in fig 3c through unsupervised clustering. The authors then use HVG methods to identify 3,012 genes and then carries out clustering based on those. To evaluate the methods the silhouette score is used, but the labels from the cESFW clustering is used as ground truth. This does not sound like a fair way to compare. Could the authors please clarify, and if needed come up with an approach where the three methods have a more level playing field if needed.

The reviewer raises a fair point regarding the comparison of cluster identities and ranked gene lists. This issue is a chicken and egg problem, in that we require a baseline to benchmark different methodologies but lack an explicitly defined ground truth. For that reason we used synthetic datasets for initial comparison.

For the human embryo data, we have presented substantial evidence that our cluster annotations are biologically coherent and consistent with prior knowledge. We therefore consider it legitimate to compare the ranked lists of Seurat, Scran and cESFW. However, we acknowledge the potential bias and have mentioned this in the "Limitations of the study" section.
In addition, we have now analysed the peripheral blood mononuclear cells (PBMC) scRNA-seq dataset that is used in the tutorial workflows of Seurat and Scran. This PBMC dataset is arguably better defined since it has more discrete populations of cells, and by using the Seurat generated cell type labels we bias the analysis towards Seurat rather than cESFW. The results show that cESFW performs comparably to Seurat and Scran, and that the cESFW ranked gene list may be more stable than Seurat and Scran. These results suggest that cESFW can be widely applicable as a suitable alternative for feature selection. We have included this analysis in the Results and as a supplemental figure (New figure, Figure S2).

5 The main cESFW.py file in the github repository is clearly well structured and commented. However, I would like to see a much better documentation so that one does not have to go through the source code to understand what functions there are and what they do. In particular, I would like to see a vignette to make it easier for others to incorporate cESFW into their workflows.

We thank the reviewer for the positive comments regarding our cESFW.py commenting. We accept that our initial submission failed to point the reader directly towards our example workflows that provide step by step, well commented vignettes for using cESFW to analyse scRNA-seq data. In our initial submission we provided 5 workflows (4 synthetic data and the human embryo data), and in

© 2024. Published by The Company of Biologists under the terms of the Creative Commons Attribution License (https://creativecommons.org/licenses/by/4.0/).

the re-submission we have added a workflow for analysing PBMC data. We have updated our cESFW Github to guide users to these example workflows (https://github.com/aradley/cESFW/tree/main).

Please note, the embryo workflow will be easily accessible through GitHub, whereas the synthetic data and PBMC workflows will be provided through a Mendeley data link (referenced in the manuscript and on our GitHub). However, the content of the Mendeley link cannot be made public until the paper is finalised, as it cannot be changed after publication. We provide a temporary public Dropbox link for the reviewers so that they may access the additional workflows (https://www.dropbox.com/scl/fo/xr5o9xm6490ftjsa55wxg/h?rlkey=maindrxwdqnirsw1en3my5qsr&dl=0).

Minor:

Why are the figures not always in order? For example, fig S10 is mentioned before fig S2 on p 6

Thank you for pointing this out; we have amended the text.

I am not sure if the indexing in eq 1 (p 18) is correct. j is both on the LHS and it is also being summed over on the RHS. Should one of these be i instead?

The indexing is correct. Each column *j* of a matrix refers to gene/feature on the RHS, and in the calculation on the RHS we take the column averages, leading to vector on the LHS that is still indexed by genes/features *j*. We have clarified this in the text.

Reviewer #2 (Significance (Required)):

The work presents a new method for feature selection in scRNAseq. Feature selection is a very important step and can have a big impact on findings. The method presented here is theoretically sound and it seems to provide interesting result when applied to early embryo development. However, as cESFW is only tested for one dataset it is unclear how well the method generalizes to other problems and datasets.

Appreciation of the utility of cESFW will grow as it is applied to more datasets. However, we would like to highlight that the human embryo dataset consists of 6 independent scRNA-seq datasets from different laboratories, and that cESFW was able to identify common and differing structure between them without any batch correction, smoothing or feature extraction. We have added to our summary that we propose cESFW may be best suited to analysis of transcriptome trajectories in time course and developmental data. However, we have also now performed comparison of Seurat, Scran and cESFW feature selection in a different context, using a reference PMBC scRNA-seq dataset. The results demonstrate that cESFW is a viable alternative for feature selection in that static system also (New figure, Figure S2).

Original submission

First decision letter

MS ID#: DEVELOP/2024/202832

MS TITLE: Branching topology of the human embryo transcriptome revealed by entropy sort feature weighting

AUTHORS: Arthur Radley, Stefan Boeing, and Austin G Smith

I have now received all the referees reports on the above manuscript, and have reached a decision. The referees' comments are appended below, or you can access them online: please go to BenchPress and click on the 'Manuscripts with Decisions' queue in the Author Area.

© 2024. Published by The Company of Biologists under the terms of the Creative Commons Attribution License (https://creativecommons.org/licenses/by/4.0/).

The overall evaluation is positive and we would like to publish a revised manuscript in Development, provided that the referees' comments can be satisfactorily addressed. Please attend to all of the reviewers' comments in your revised manuscript and detail them in your point-by-point response. If you do not agree with any of their criticisms or suggestions explain clearly why this is so. If it would be helpful, you are welcome to contact us to discuss your revision in greater detail. Please send us a point-by-point response indicating your plans for addressing the referees' comments, and we will look over this and provide further guidance.

Reviewer 1

Advance summary and potential significance to field

This manuscript will clearly be an important contribution to the fields of scRNAseq analysis, which is a major method to understand developmental biology.

Comments for the author

I thank the authors for their carefull revision.

Reviewer 2

Advance summary and potential significance to field

Radley, Boeing, and Smith propose a new method for identifying highly variable genes (HVGs) in single cell RNA sequencing data as an extension of their previous work with Entropy Sorting. In this paper, they outline the computational basis for modifying Entropy Sort Feature Weighting (ESFW) to work with continuous variables as found in single cell gene expression. The cESFW algorithm is validated on synthetic data and through a re-analysis of publicly available early human embryo development datasets. From the synthetic data, the authors show that cESFW outperforms both Seurat's and Scran's methods, with the most improvement in synthetic datasets with high expression of ubiquitously expressed genes. This is a notable achievement, as it has been established that existing HVG selection methods struggle in those scenarios. The re-analysis of human embryo data with cESFW revealed two new transitory cell clusters and provides support for the presence of two lineage bifurcations in early human development. Previous single cell analyses have been unable to definitively show this, and the authors provide evidence that using cESFW for feature selection is able to differentiate these intermediate populations more accurately. The author's initial goal of not modifying the count matrix is quite desirable, and they successfully demonstrate a valid analysis driven by their cESFW method.

Comments for the author

Major Comments:

- There is a heavy reliance on analysis of UMAP projections, including for cell type label transfer. This should be done in the higher dimensional space rather than the embedding, using existing tools such as CellTypist, SingleR, or scANVI. Minimizing the UMAP analysis in other areas by using differential expression, trajectory analysis, or other methods would also strengthen the argument.
- RNA velocity is a powerful tool, but the noted lack of unspliced counts for all datasets as well as numerous potential pitfalls make its use here not ideal. Using a trajectory inference method like Palantir, Slingshot, or PAGA could complement or supersede the RNA velocity results and show a more robust trajectory.
- In the comparisons to Scran and Seurat, the authors do not follow the recommended workflows and avoid using PCA prior to UMAP dimensionality reduction. Although this is to be comparable to cESFW, this weakens the comparison results as it is not the correct way of using the existing tools.

Minor Comments:

© 2024. Published by The Company of Biologists under the terms of the Creative Commons Attribution License (https://creativecommons.org/licenses/by/4.0/).

- How scalable is cESFW? Consider including an analysis with computation times for varying cell and gene numbers.
- Edit the installation instructions on the package github page. Package installation instructions should not instruct the user to run `python setup.py install`. Packages should be published on PyPI and installable with `pip install`.
- The shiny app linked in the paper is not accessible (Proxy Error – Error reading from remote server).

First revision

Author response to reviewers' comments

Reviewer 1 Advance summary and potential significance to field

This manuscript will clearly be an important contribution to the fields of scRNAseq analysis, which is a major method to understand developmental biology.

Reviewer 1 Comments for the author

I thank the authors for their careful revision.
We appreciate these remarks.

***** Reviewer 2 Advance summary and potential significance to field

Radley, Boeing, and Smith propose a new method for identifying highly variable genes (HVGs) in single cell RNA sequencing data as an extension of their previous work with Entropy Sorting. In this paper, they outline the computational basis for modifying Entropy Sort Feature Weighting (ESFW) to work with continuous variables as found in single cell gene expression. The cESFW algorithm is validated on synthetic data and through a re-analysis of publicly available early human embryo development datasets. From the synthetic data, the authors show that cESFW outperforms both Seurat's and Scran's methods, with the most improvement in synthetic datasets with high expression of ubiquitously expressed genes. This is a notable achievement, as it has been established that existing HVG selection methods struggle in those scenarios. The re-analysis of human embryo data with cESFW revealed two new transitory cell clusters and provides support for the presence of two lineage bifurcations in early human development. Previous single cell analyses have been unable to definitively show this, and the authors provide evidence that using cESFW for feature selection is able to differentiate these intermediate populations more accurately. The author's initial goal of not modifying the count matrix is quite desirable, and they successfully demonstrate a valid analysis driven by their cESFW method.

Reviewer 2 Comments for the author

Major Comments:

-There is a heavy reliance on analysis of UMAP projections, including for cell type label transfer. This should be done in the higher dimensional space rather than the embedding, using existing tools such as CellTypist, SingleR, or scANVI. Minimizing the UMAP analysis in other areas by using differential expression, trajectory analysis, or other methods would also strengthen the argument.

We understand the reviewer's suggestion to perform label transfer directly in the high dimensional space. However, this is problematic for these human embryo scRNA-seq datasets. The combined data contain only 2400 cells, far fewer than is typically required for machine learning techniques such as those suggested. This makes it difficult to obtain robust results that are consistent with the literature, due to factors such as high levels of noise, class imbalance between reference labels

© 2024. Published by The Company of Biologists under the terms of the Creative Commons Attribution License (https://creativecommons.org/licenses/by/4.0/).

(over-representation of trophoblast lineage cells), and batch affects between datasets and sequencing platforms.

However, we agree that UMAP label transfer should be complemented with an alternative methodology. We therefore calculated correlation distances in the high dimensional gene space. To do so, we took the human embryo data and created pseudo-bulk samples according to our cell type annotations. We then determined their correlation distance with the cultured PSC scRNA-seq samples. The embryo cell types that each of the cultured PSCs are most similar to in the high dimensional gene space match closely the cell type label transfer predictions in the UMAP space. These results are summarised in a new supplemental figure (Figure S8) and are discussed in the main text.

-RNA velocity is a powerful tool, but the noted lack of unspliced counts for all datasets as well as numerous potential pitfalls make its use here not ideal. Using a trajectory inference method like Palantir, Slingshot, or PAGA could complement or supersede the RNA velocity results and show a more robust trajectory.

We have now performed trajectory analysis with the popular scRNA-seq package, STREAM (https://www.nature.com/articles/s41467-019-09670-4). The results complement the RNA velocity results, showing smooth trajectories and clear branching points along the differentiation trajectory. We have added these results to Figure S6 and mention them alongside the RNA velocity results in the main text.

-In the comparisons to Scran and Seurat, the authors do not follow the recommended workflows and avoid using PCA prior to UMAP dimensionality reduction. Although this is to be comparable to cESFW, this weakens the comparison results as it is not the correct way of using the existing tools.

We acknowledge the reviewer's point and have adjusted the heading of this section to more neutral language: "Comparison of cESFW with HVG selection techniques on human embryo data". In the text we now state explicitly: "For direct comparison, we omitted prior PCA selection for the Scran and Seurat UMAPs, because PCA reduction is not part of the cESFW workflow." Nonetheless, we have to point out that a series of publications have used the conventional scRNA-seq workflows to analyse these human scRNA-seq data and have been unable to resolve high resolution cell type identities.

Minor Comments:

-How scalable is cESFW? Consider including an analysis with computation times for varying cell and gene numbers.

cESFW can be run on datasets of 10's of thousands of cells and genes on a laptop with 32GB RAM. We will soon be updating the software to be faster and more memory efficient. Once this is complete, we will add scalability and computational run times to our Github repository.

-Edit the installation instructions on the package github page. Package installation instructions should not instruct the user to run `python setup.py install`. Packages should be published on PyPI and installable with `pip install`.

We have intentionally provided the software outside of the PyPi/pip install framework so that the source code can be more easily accessed and interrogated. We believe this is useful for those who would like to gain a better understanding of how Entropy Sorting is being implemented since it is a relatively new mathematical framework for data analysis.

-The shiny app linked in the paper is not accessible (Proxy Error – Error reading from remote server).

We apologise for the link not being accessible to the reviewer. Occasionally the Francis Crick Institute servers are not accessible, but we assure the reviewers and editor that the link is working and will be maintained.

© 2024. Published by The Company of Biologists under the terms of the Creative Commons Attribution License (https://creativecommons.org/licenses/by/4.0/).

Second decision letter

MS ID#: DEVELOP/2024/202832

MS TITLE: Branching topology of the human embryo transcriptome revealed by entropy sort feature weighting

AUTHORS: Arthur Radley, Stefan Boeing, and Austin G Smith
ARTICLE TYPE: Research Article

Thank you for sending your manuscript to Development through Review Commons.

I am happy to tell you that your manuscript has been accepted for publication in Development, pending our standard ethics checks.

Reviewer 1

Advance summary and potential significance to field

As stated previously, this manuscript is an important contribution.

Comments for the author

I thank you for appropriately addressing all the revision rounds.

Reviewer 2

Advance summary and potential significance to field

Radley, Boeing, and Smith propose a new method for identifying highly variable genes (HVGs) in single cell RNA sequencing data as an extension of their previous work with Entropy Sorting. In this paper, they outline the computational basis for modifying Entropy Sort Feature Weighting (ESFW) to work with continuous variables as found in single cell gene expression. The cESFW algorithm is validated on synthetic data and through a re-analysis of publicly available early human embryo development datasets. From the synthetic data, the authors show that cESFW outperforms both Seurat's and Scran's methods, with the most improvement in synthetic datasets with high expression of ubiquitously expressed genes. This is a notable achievement, as it has been established that existing HVG selection methods struggle in those scenarios. The re-analysis of human embryo data with cESFW revealed two new transitory cell clusters and provides support for the presence of two lineage bifurcations in early human development. Previous single cell analyses have been unable to definitively show this, and the authors provide evidence that using cESFW for feature selection is able to differentiate these intermediate populations more accurately. The author's initial goal of not modifying the count matrix is quite desirable, and they successfully demonstrate a valid analysis driven by their cESFW method.

Comments for the author

I thank the authors for their thorough response. The revision and author comments addressed all major and minor comments.

© 2024. Published by The Company of Biologists under the terms of the Creative Commons Attribution License (https://creativecommons.org/licenses/by/4.0/).
